# The impact of climate and vegetation on the riverside architecture of the Brazilian Amazon

**DOI:** 10.1038/s41598-026-50315-6

**Published:** 2026-07-27

**Authors:** Maria Cristina Celuppi, Emerson Galvani, Adriana Cristina da Silva Nunes, Graziela Tosini Tejas, Reginaldo Martins da Silva de Souza, Marlon Resende Faria, Glenda Natalia Passos, João Paulo Assis Gobo

**Affiliations:** 1 University of São Paulo, São Paulo, Brazil; 2 Federal University of Rondônia, Porto Velho, Brazil; 3 Federal Institute of Rondônia, Porto Velho, Brazil

**Keywords:** Climate, Vegetation, Riverside architecture, Traditional riverside knowledge, Environmental conservation, Amazon, Climate sciences, Ecology, Ecology, Environmental sciences

## Abstract

This research aimed to investigate the impact of vegetation conservation on the local climate and architectural production of two riverside communities in one of the capitals of the Brazilian Amazon, one close to and the other far from the urban area. Based on the inductive-exploratory method, we collected primary climatological data in the external area and inside the dwellings in both communities (October 2022 to May 2023), developed analyses of the Normalized Difference Vegetation Index (NDVI) and Land Surface Temperature (LST) (August 2022 to May 2023), and finally collected architectural information from the dwellings investigated. Our results show that the climatological data from the two communities are statistically different, being more favorable to comfort in the community with greater vegetation conservation. Analysis of remote sensing images showed that vegetation density in this community indicates a strong relationship with vegetative regeneration capacity after the most critical dry months of the period investigated. Regarding thermal comfort inside dwellings, the data showed that, both through statistical analysis and the thermal comfort model adopted, the combined effect of building materials, vegetation cover, and environmental context favors better thermal comfort conditions. The results presented point to the importance of vegetation conservation, which positively influenced the local climate and thermal comfort of riverside dwellings. In addition, our results encourage the perpetuation of traditional knowledge that adapts architecture to the forest.

## Introduction

The environmental, social, and health conditions in riverside communities in the Brazilian Amazon are directly influenced by development projects in the region^1^. Therefore, understanding the reality of these communities through the influence of climate, environmental conservation, and vernacular construction processes that shape the spatial distribution of communities and the ways of living of riverside dwellers is an important tool in the socio-environmental development of these populations.

The riverside architecture of this region is characterized by a combination of distinct parameters, influenced primarily by the cycle of river floods and ebbs, local climatic conditions, conservation of these regions, local cultural aspects, materials available in the region, and the notion of home and local aesthetics^2^. However, it is expected that the combination of these parameters will result in dwellings that are compatible with the environment, the local climate, and the water cycles. Nevertheless, Amazonian communities are not homogeneous^3^ in terms of culture, social environment, and ethnicity, reflecting a great diversity that results in spatial configurations with non-traditional buildings, introducing materials that are not always suitable for those climatic conditions. Previous studies^4,5^ also investigated riverside dwellings in this region and observed that local dwellings, although influenced by the climatic context and the river cycle, present critical scenarios for human comfort. However, simple construction strategies can promote considerable improvement in the comfort conditions of this population.

**Fig. 1:**
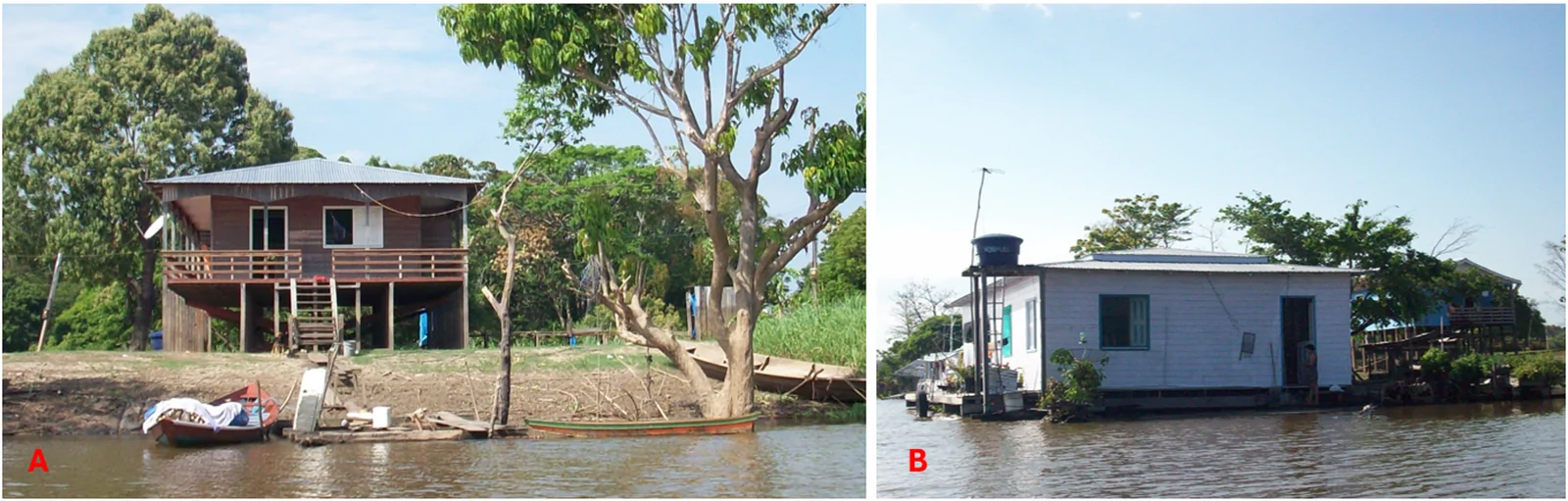
Stilt and floating dwellings in the Brazilian Amazon region. (Source: The authors)

In the Amazon region, there are two predominant types of dwellings that make up the local communities: *stilt houses* (Fig. 1A), which are built on river floodplains, elevated on stilts and suspended by supports, built in this way so that they are not flooded during periods of high water, and also the *floating houses* (Fig. 1B), which have the same characteristics as stilt houses, but are built on wooden logs, which allow them to move and float according to the river cycles^2,4–6^.

When addressing the context of riverside communities, one cannot fail to associate architectural aspects with the availability of vegetation, which, in turn, has a direct impact on health and is related to various benefits for the physical and mental health of the population, since environments with well-distributed vegetation contribute to a significant improvement in overall health, stress management, incidence of certain diseases, increased life expectancy, among many other factors already referenced in the literature^7–13^.

With a view to the United Nations (UN) 2030 Agenda for the implementation of public policies that meet the Sustainable Development Goals (SDGs), conducting scientific research that seeks to understand the scenario and complexities of Amazonian riverside communities is still a gap that has been under investigated and explored by researchers. This research could address some of the UN Sustainable Development Goals (SDGs), such as Goal 11—Sustainable Cities and Communities, which seeks to “make cities and human settlements inclusive, safe, resilient, and sustainable,” and Goal 15—Life on Land, which seeks to “protect, restore and promote the sustainable use of terrestrial ecosystems, sustainably manage forests, combat desertification, halt and reverse land degradation and halt biodiversity loss.” Thus, understanding the spatial dynamics of riverside areas through the relationship between climate, vegetation, and vernacular architecture presents itself as an important line of research to be investigated.

Given this, with the municipality of Porto Velho, capital of the state of Rondônia, as a representative study site for the Brazilian Amazon, and considering the lack of academic studies that integrate climate, vegetation, and architecture in riverside areas of the state and the country, this research aims to:

1. Understand the construction aspects of riverside dwellings, given the local climatic and environmental conditions, in communities located in areas of greater and lesser landscape conservation;
2. Understand and discuss the impact of local vegetation on the comfort of these communities, taking into account the level of conservation of the study sites;
3. Discuss the role of environmental conservation and traditional architecture in these communities;

### Characterization of the study area

**Fig. 2:**
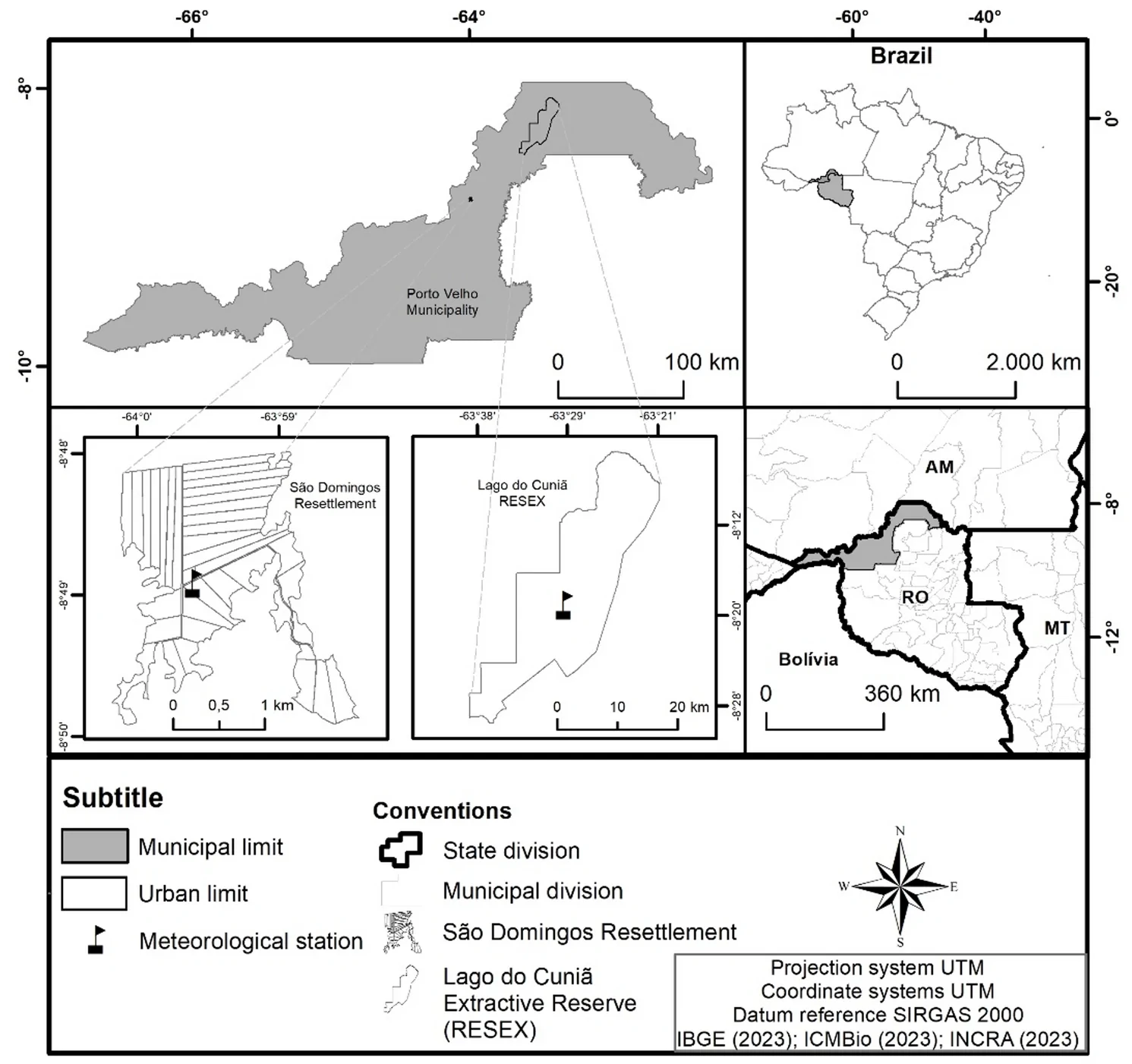
Study area location map (created using ArcMap 10.5 software: https://www.arcgis.com/index.html). (Org.: The authors)

During the exploratory period of the Amazon region, the state of Rondônia was part of government and political goals that directly influenced the social and spatial formation of this region, from which the state capital, Porto Velho, emerged during the construction of the Madeira-Mamoré Railway Station on the banks of the Madeira River, around 1907^14,15^. Part of the Amazon biome, with the central landmark of the municipality located at latitude 8° 45′ 43″ South and longitude 63° 54′ 7″ West, Porto Velho (Fig. 2) has a territory of 34,090.952 km² and has 460,413 inhabitants^15^.

In the current literature, there are no studies conducted in rural areas of Porto Velho that aim to investigate comfort, especially those that address the context of local riverside communities. To understand the social dynamics of these areas, there are currently reports from organizations and institutes, such as the Amazon Riverside Population Support Center (NAPRA) and the Chico Mendes Institute for Biodiversity Conservation (ICMBio). The research available for academic consultation on this topic has been conducted in the urban area of the capital, pointing out the weaknesses of the urbanization process and the expansion of agriculture and livestock farming that has occurred and continues to occur to the detriment of green areas and the natural landscape.

As for the climate, the municipality of Porto Velho has an “Aw” climate, tropical rainy with a climatological average air temperature during the coldest month above 18 °C (megathermal), a well-defined dry period during the winter in the Southern Hemisphere, and rainfall indices below 50 mm/month^16–18^. In relation to average annual precipitation, pronounced seasonality is observed for the study area, with high values between November and May and low values between June and September^19,20^. The same seasonality is observed in relation to air temperature and relative humidity^21–23^, with higher humidity from December to May and a drier period from June to November. The air temperature is higher between June and October, when the lowest temperatures are also observed due to the astronomical winter and the arrival of polar systems, causing cold spells^24^.

To better understand the local riverside scenario, this research sought to understand the climatic, vegetative, and architectural scenario of two riverside communities within the limits of the municipality of Porto Velho, the Lago do Cuniã Extractive Reserve (RESEX) and the São Domingos Resettlement.

**Fig. 3:**
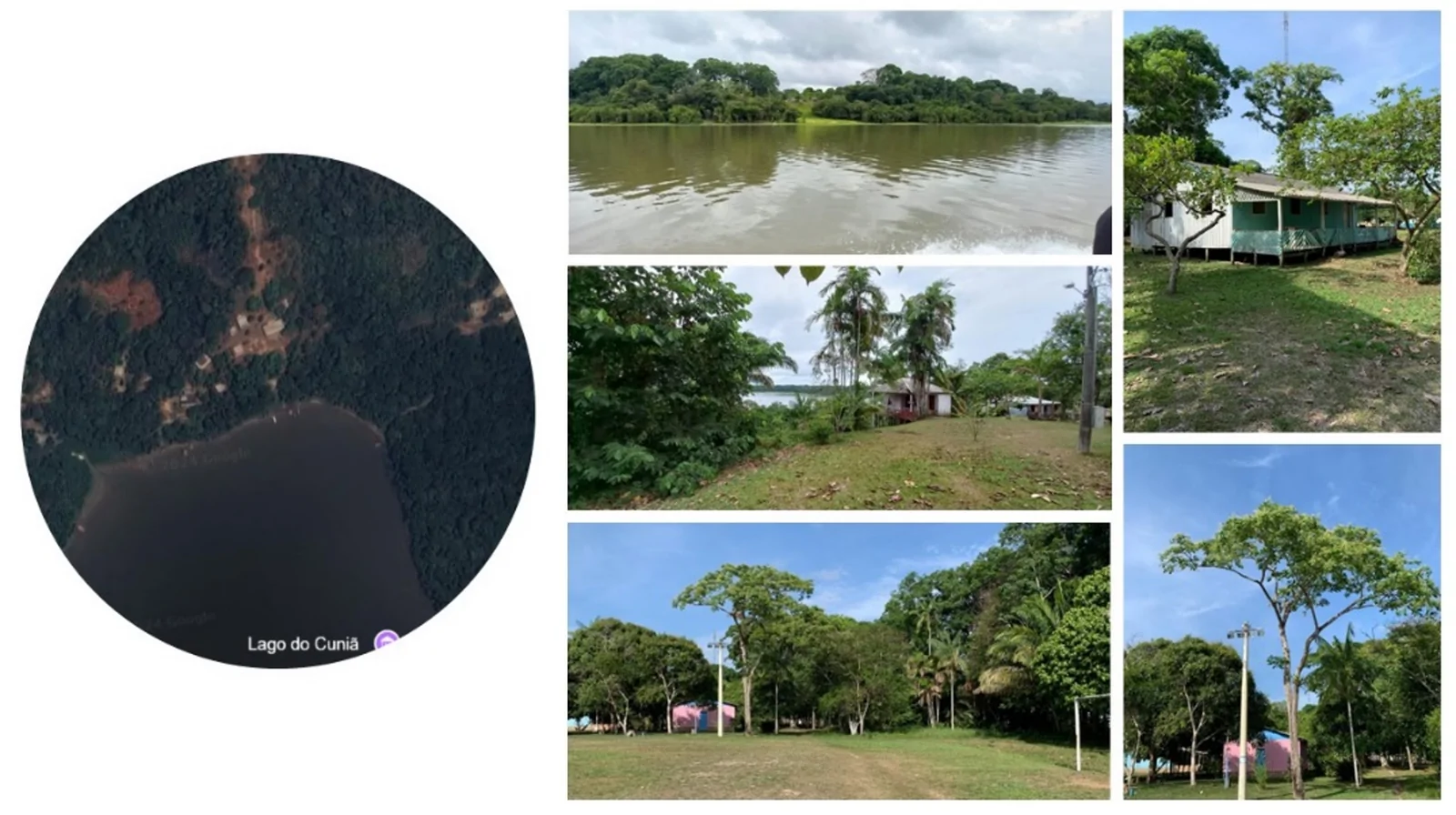
Lago do Cuniã Extractive Reserve (RESEX) (created using Google Maps: https://www.google.com/maps/place/PortoVelho/RO). (Source: The authors)

The Lago do Cuniã Extractive Reserve (Figs. 2 and 3), popularly known as RESEX Lago do Cuniã, is a federal conservation unit (CU) for sustainable use, located on the left bank of the Madeira River in the rural area of Porto Velho (about 130 km from the urban center), which aims to conserve the natural environment and ensure the sustainable use of natural resources, as well as to conserve these resources^25^.

**Fig. 4:**
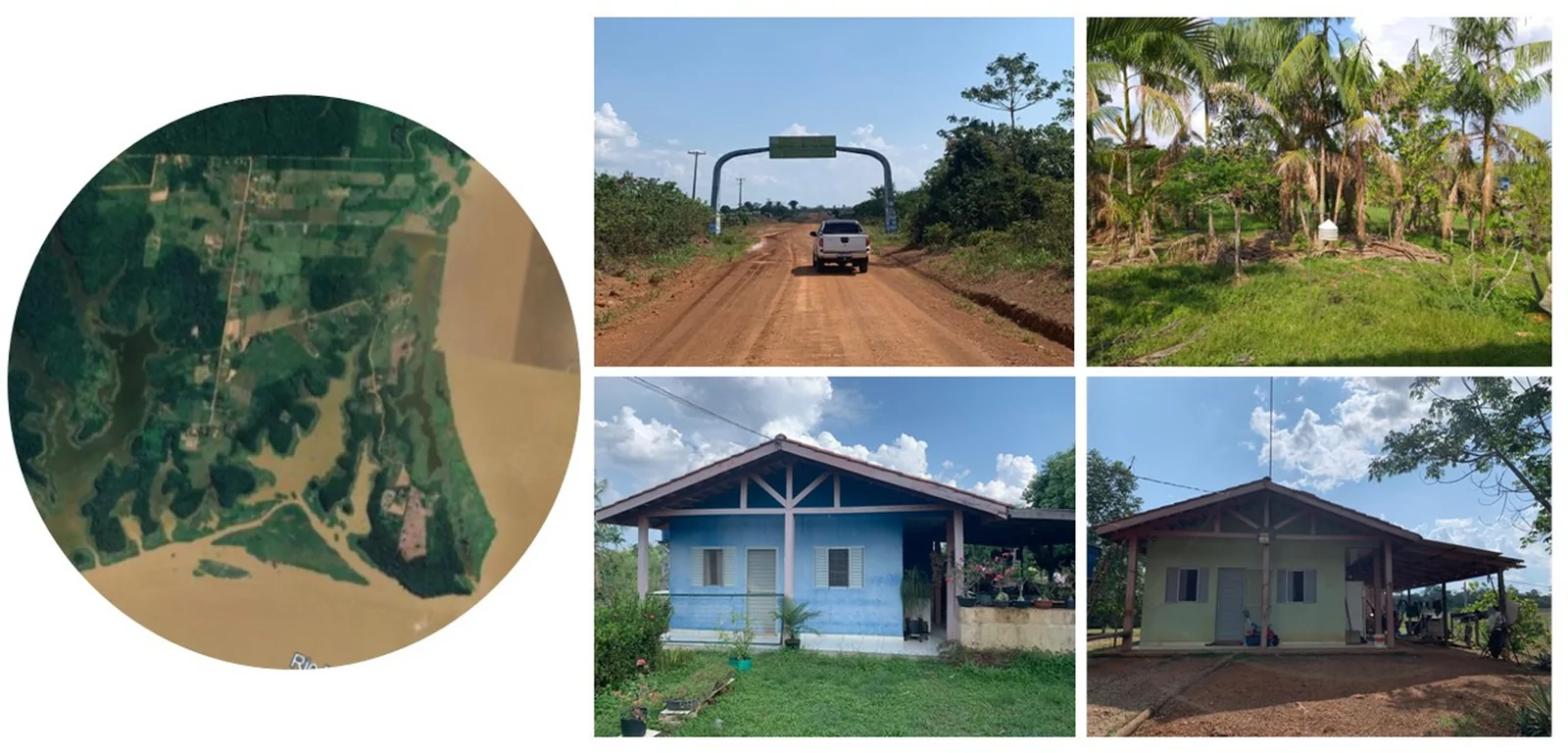
São Domingos Resettlement (created using Google Maps: https://www.google.com/maps/place/PortoVelho/RO). (Source: The authors)

The construction of the Santo Antônio Hydroelectric Plant on the Madeira River, a result of the Growth Acceleration Plan (PAC) in 2006, created resettlements for families whose lands were occupied by the creation of the lake and construction site for the plant, forming the São Domingos Resettlement (Figs. 2 and 4), on the left bank of the Madeira River. According to the Porto Velho administration, the São Domingos Resettlement, located 22 km from the urban area of Porto Velho, is considered a model for diversification in family farming, which ensures the maintenance of its economic activities. Land use and occupation in the Resettlement is essentially characterized by the presence of extensive agriculture and livestock farming, mixed with preserved areas.

## Method

To elucidate the objectives of this research, we opted for the “inductive-exploratory” method, based on the inductive character through an empiricist method, which considers knowledge as based on experience, where generalization derives from observations of concrete reality cases, elaborated from particular findings, as well as the exploratory character, which aims to provide greater familiarity with a particular problem, involving bibliographic research and analysis of examples, generally taking the form of research^26^.

To this end, the research will be developed in four stages that will support the objectives presented here: collection and analysis of climate data; analysis of vegetation; observation of construction techniques; and critical analysis and discussion.

### Collection and analysis of climatological data

**Fig. 5:**
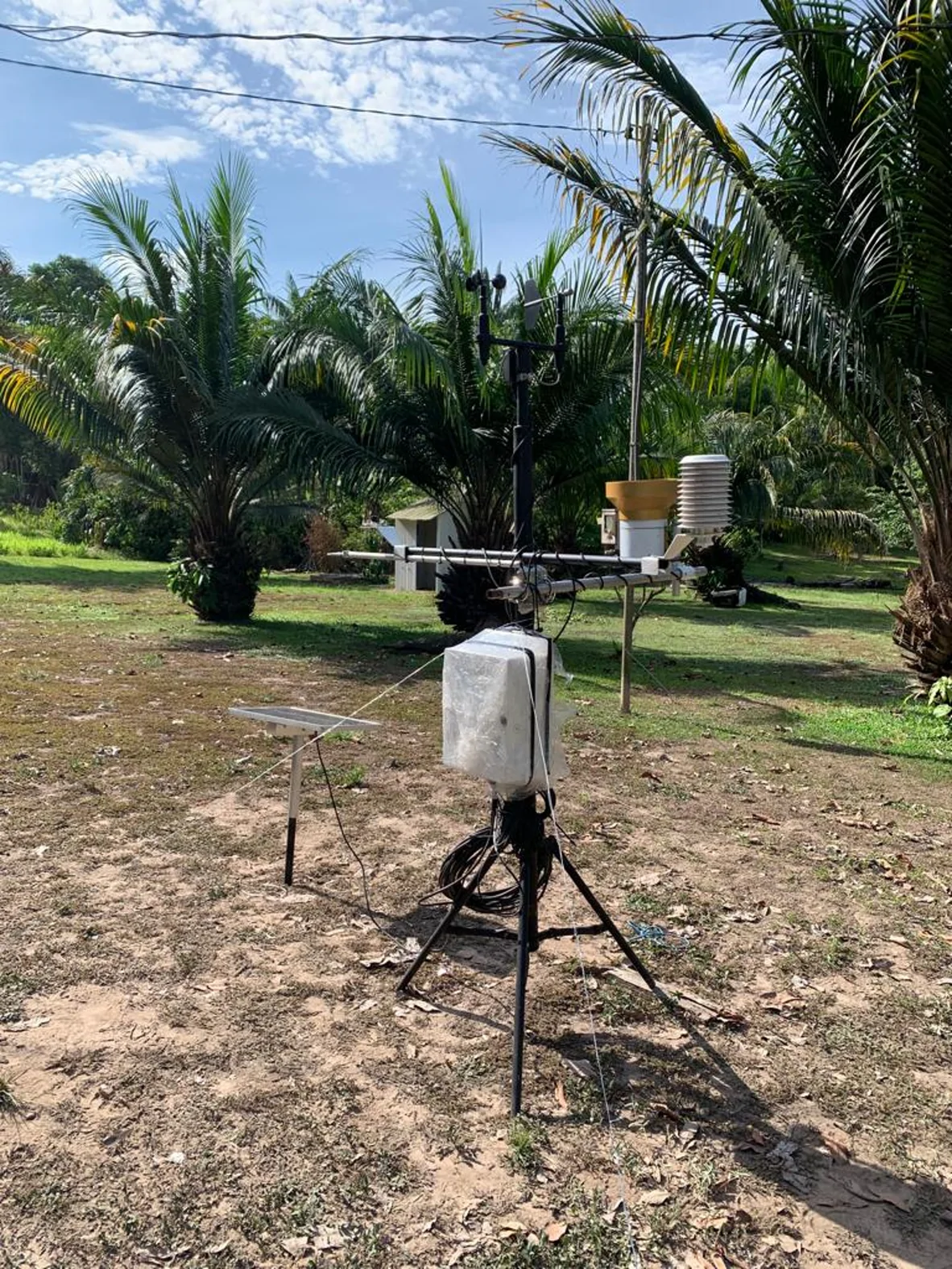
Campbell CR-1000 weather station, installed in the Lago do Cuniã Extractive Reserve. (Source: The authors)

For the data collection process, fieldwork was carried out at RESEX, where a Campbell CR-1000 weather station (Fig. 5) was installed to collect climate data outside the dwellings. It has a data logger, tipping bucket rain gauge, global radiation sensor, air temperature and humidity sensor, wind speed and direction sensor, and gray globe thermometer.

To represent outdoor data on the Resettlement, data provided by the Santo Antônio Hydroelectric Plant weather station, located near the Resettlement, was used.

Therefore, climatological data will be presented for a community in a conservation area and another that, due to its location near the urban perimeter, is influenced by the urban area of the capital of Rondônia.

The data collected were tabulated in a Microsoft Excel^®^ spreadsheet, with hourly, daily, and monthly averages calculated. To determine whether the values of a measured variable, denoted as “a,” are consistently higher than in another location, denoted as “b,” it was necessary to analyze the frequency with which the measurements in ‘a’ exceed those in “b” at the same time. In addition, the proportion of times that the measurements were equal was considered.

To assess the difference between the measurements, that is, to quantify the magnitude of the variation between them over time, the difference between the measurements of “a” and “b” was calculated for each corresponding time, thus obtaining the average of this difference.

Finally, a paired t-test was performed to statistically verify whether the series show significant differences. This test allows us to state, with a certain level of significance, whether the series averages are distinct, regardless of possible similarities in behavior over time. The frequency can be calculated as follows, for example:

$${\mathrm{1}}00 - \left( {{\mathrm{R}}{{\mathrm{H}}_{{\mathrm{resettlement}}\_{\mathrm{outdoor}}}}>{\mathrm{R}}{{\mathrm{H}}_{{\mathrm{resex}}\_{\mathrm{outdoor}}}}} \right) - \left( {{\mathrm{R}}{{\mathrm{H}}_{{\mathrm{resettlement}}\_{\mathrm{outdoor}}}}={\mathrm{R}}{{\mathrm{H}}_{{\mathrm{resex}}\_{\mathrm{outdoor}}}}} \right)$$

where RH is the relative humidity (analyzed variable).

Subtraction by 100 is necessary to reverse the relationship and, additionally, to discount cases where the values were equal.

Finally, the paired t-test was used, whose hypotheses are:

- Null hypothesis (H_0_): the series averages have the same value;
- Alternative hypothesis (H_1_): the series averages have different values.

To discard the null hypothesis, it was then defined that the p-value must be less than the significance level Alpha = 0.01.

To analyze the indoor data of local dwellings, HT-500 Instrutherm data loggers were installed in two dwellings, one in the RESEX and the other in the Resettlement, which collected data on temperature and relative humidity. We decided to investigate two dwellings, given the availability of the measuring equipment we had to conduct the study.

The analysis of the indoor data of the dwellings was conducted in the same way as the outdoor data mentioned above and using the adaptive comfort model for Southeast Asia proposed by Nguyen et al.^28^, who developed an adaptive comfort model for naturally ventilated buildings in hot and humid regions of Southeast Asia, a region with the same climate type as Porto Velho, RO. This model was previously used by Celuppi^4^ and Celuppi et al.^5^ for the municipality of Manacapuru, Amazonas, and proved to be efficient in analyzing comfort in this region.

The model calculates the indoor comfort temperature as a linear function of the average monthly outdoor temperature, considering that during the course of a month outdoor temperature variations in hot and humid climates during the day are minimal, which validates the use of the outdoor monthly average for the calculation of comfort temperature^27–30^.

The comfort equation obtained by^28^ is very close to the equations provided by ASHRAE 55-2004 and the European standard EN15251, which points to a convergent trend in adaptive comfort studies^28^. Previous studies^31,32^ used this model and stated that it has a lower overall error in comfort prediction when compared to EN15251 and ASHRAE 55-2004.

The model considers the monthly average outdoor air temperature as the basis for obtaining the indoor comfort temperature and is calculated using the following equation:

$${{\mathrm{T}}_{{\mathrm{comfort}}}}=0.{\mathrm{341}}*{{\mathrm{T}}_{{\mathrm{a}}\_{\mathrm{outdoor}}}}+{\mathrm{18}}.{\mathrm{83}}$$

where T_comfort_ is the Comfort temperature; T_a_ is the Outdoor air temperature.

The data obtained through this equation are subsequently analyzed and discussed in relation to the other variables analyzed in the study.

### Vegetation analysis using Normalized Difference Vegetation Index (NDVI) and Land Surface Temperature (LST)

#### Vector cartographic data

The vector cartographic data used in this research were the shapefiles of the São Domingos Resettlement perimeter (REA São Domingos), through the base made available by the Santo Antônio Hydroelectric Plant and INCRA (https://certificacao.incra.gov.br/csv_shp/export_shp.py), as well as the shapefile of the Cuniã Extractive Reserve (RESEX Cuniã), made available by ICMBio (https://www.gov.br/icmbio/pt-br/assuntos/dados_geoespaciais/mapa-tematico-e-dados-geoestatisticos-das-unidades-de-conservacao-federais), the municipal and state boundaries database by IBGE and the GeoPortal platform of Porto Velho (https://geoportal.portovelho.ro.gov.br/). The Universal Transverse Mercator (UTM) projection system and the SIRGAS 2000 datum were also adopted in zone 20 S.

#### Acquisition and processing of satellite images

In this stage of the research, remote sensing techniques were used for digital image processing. Images from LANDSAT 8 and 9 satellites (OLI and TIRS sensors) from Collection 2 Level-1, acquired free from the United States Geological Survey (USGS), available on the institution’s website (https://earthexplorer.usgs.gov/), were used. The LANDSAT 8 and 9 (OLI and TIRS) data per orbit/point and time series are described and organized according to Table 1.

**Table 1:** Information regarding the images used for each study area. Org.: The authors

Study areas	Year	Date	Satellite/sensor	Orbit/point	Period
REA São Domingos and RESEX Cuniã	2022	08/12	Landsat 09—OLI and TIRS	232/66	Dry
	2022	09/05	Landsat 08—OLI and TIRS		Dry
	2023	05/19	Landsat 08—OLI and TIRS		Rainy/dry transition
	2023	06/28	Landsat 09—OLI and TIRS		Dry
	2023	07/22	Landsat 08—OLI and TIRS		Dry
	2023	08/07	Landsat 08—OLI and TIRS		Dry
	2023	09/08	Landsat 08—OLI and TIRS		Dry
	2023	10/18	Landsat 09—OLI and TIRS		Dry/rainy transition
	2024	06/06	Landsat 08—OLI and TIRS		Dry
	2024	07/16	Landsat 09—OLI and TIRS		Dry
	2024	08/09	Landsat 08—OLI and TIRS		Dry

For the digital processing of LANDSAT 8 and 9 images (OLI and TIRS), both for the calculation of NDVI and LST, ArcMap 10.5 software was used, where they were redesigned and cropped to the study area boundary and then corrected for radiance and reflectance. These procedures were performed based on^33^. Software URL: https://www.arcgis.com/index.html.

#### Normalized Difference Vegetation Index (NDVI) and Land Surface Temperature (LST)

After redesigning and correcting the images of Bands 4 and 5 of the Landsat 8 and 9 OLI sensor, the files were separated into raster using the tools of the ArcMap 10.5 software “ArcToolBox” (Spatial Analyst Tools – Map Algebra – Raster Calculator) and the NDVI was calculated using the following equation:

$${\mathrm{NDVI}}=({\mathrm{Band}}\;{\mathrm{5}}--{\mathrm{Band}}\;{\mathrm{4}})/({\mathrm{Band}}\;{\mathrm{5}}+{\mathrm{Band}}\;{\mathrm{4}})$$

Using the redesigned and corrected Landsat 8 and 9 Band 10 images, the land surface temperature (LST) values in Kelvin were obtained using the equation below, which were then converted to °C.

$$Ts=\left( {\frac{{K2}}{{{\mathrm{ln}}\left( {\frac{{K1}}{{L\lambda }}+1} \right)}}} \right)$$

where ST is the Surface Temperature (K); L λ is the Spectral radiance at the top of the atmosphere (W/m²*srad*µm); K1 is the Band-specific thermal conversion constant; K2 is the Band-specific thermal conversion constant.

From these data, NDVI and LST maps were created, which are presented later in the results of this study.

### Observation of construction aspects

Parallel to the installation of data collection equipment in both study locations, the construction techniques of local dwellings were observed in an exploratory manner, with sketches focusing on the construction method, materials used, positioning of the dwelling in relation to the terrain, and comfort strategies used by local residents. These sketches were redrawn with the aid of Revit (2023) software for better presentation of information and qualitative analysis.

**Fig. 6:**
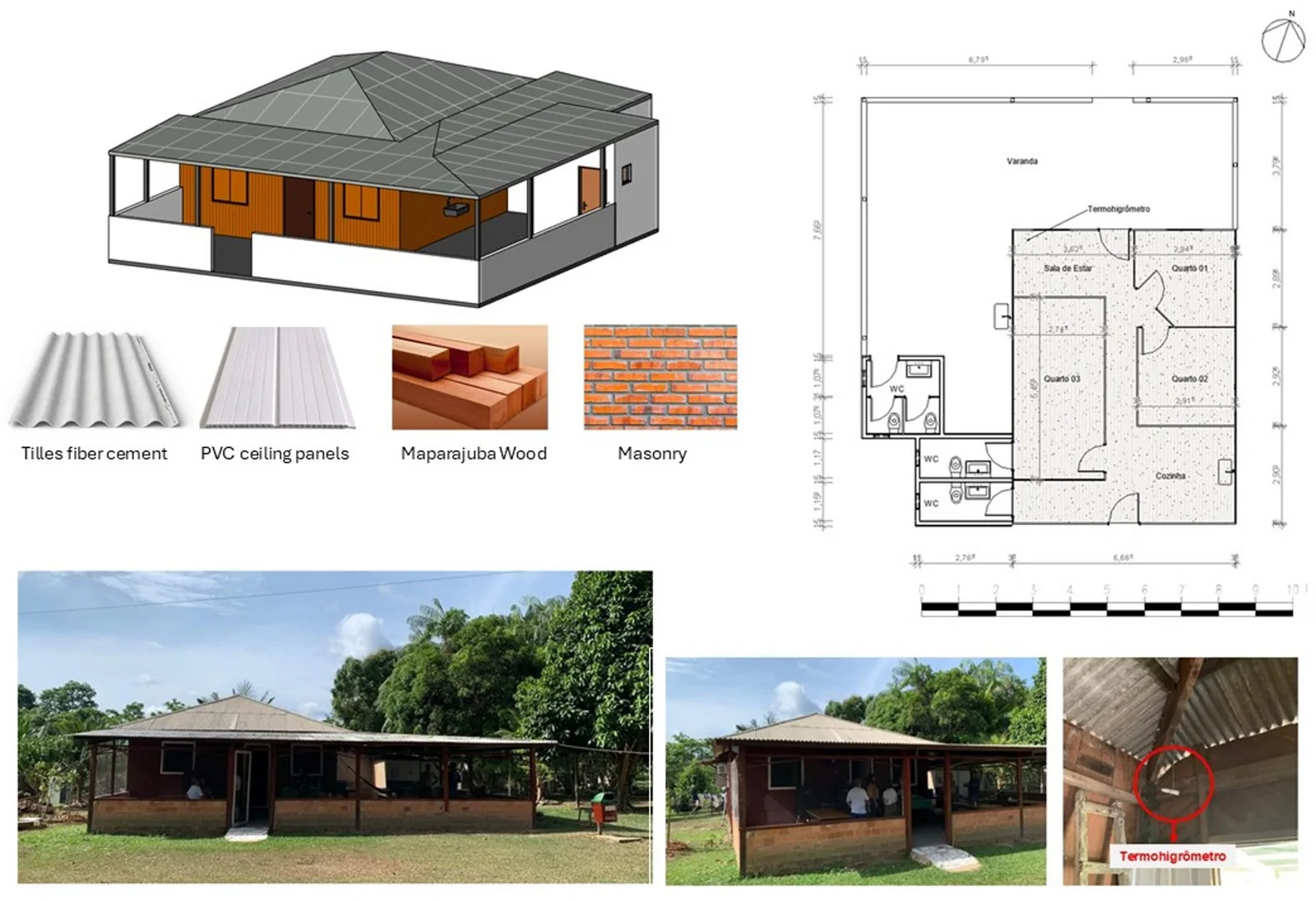
Construction aspects of the dwelling analyzed in RESEX. (Source: The authors)

As the RESEX research subject, a mixed wood and masonry house was chosen (Fig. 6). Facing north-northwest (NNW), the inside of the house has a living room, kitchen, three bedrooms, one of which is a suite, a guest bathroom and a large balcony with another guest bathroom. The rooms used for long periods of time, such as bedrooms, kitchen, and living room, are predominantly made of wood. The bathrooms and balconies are made of masonry. The roof is made of fiber cement tiles and the window frames are made of wood.

Rooms 01, 02, and 03 are the only rooms with PVC ceiling panels, and of these, rooms 01 and 03 have air conditioning units, which is why ceiling panels were used. The other rooms do not have ceilings, which is common in traditional riverside dwellings. To verify the impact of the climate on typically traditional dwellings, a data logger was installed to collect data on temperature and relative humidity in the living room, which is not air-conditioned, as shown in Fig. 6.

**Fig. 7:**
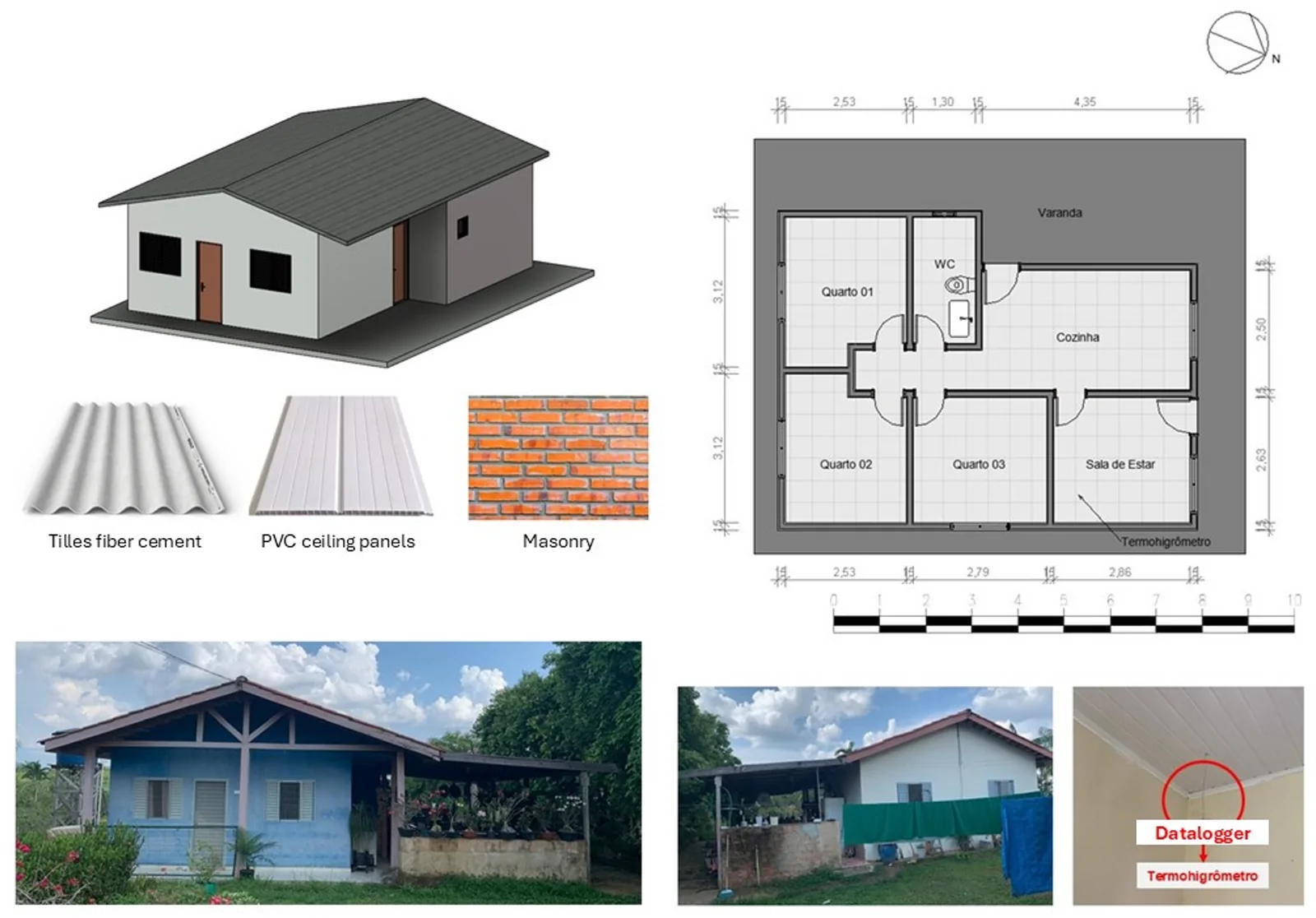
Construction aspects of the dwelling analyzed in the resettlement. (Source: The authors)

A standard masonry dwelling was used as the object of study for the Resettlement (Fig. 7). Facing north, the house has a living room, kitchen, bathroom and three bedrooms, one of which has air conditioning. The roof is made of fiber cement tiles and the window frames are metal. All the dwellings delivered in the resettlement have the same architectural plan, however, some of them have been extended by the owners.

### Critical analysis and discussion

Finally, based on the results obtained through the analysis of the collected data, a general analysis and discussion will be made on the construction process of the dwellings in relation to the local climate and considering the role of vegetation in the way of life of the riverside communities analyzed.

## Presentation of results

### Collection of climatological data

Climatological data were collected simultaneously in the Lago do Cuniã RESEX and the São Domingos Resettlement from October 15, 2022, to May 20, 2023.

**Fig. 8:**
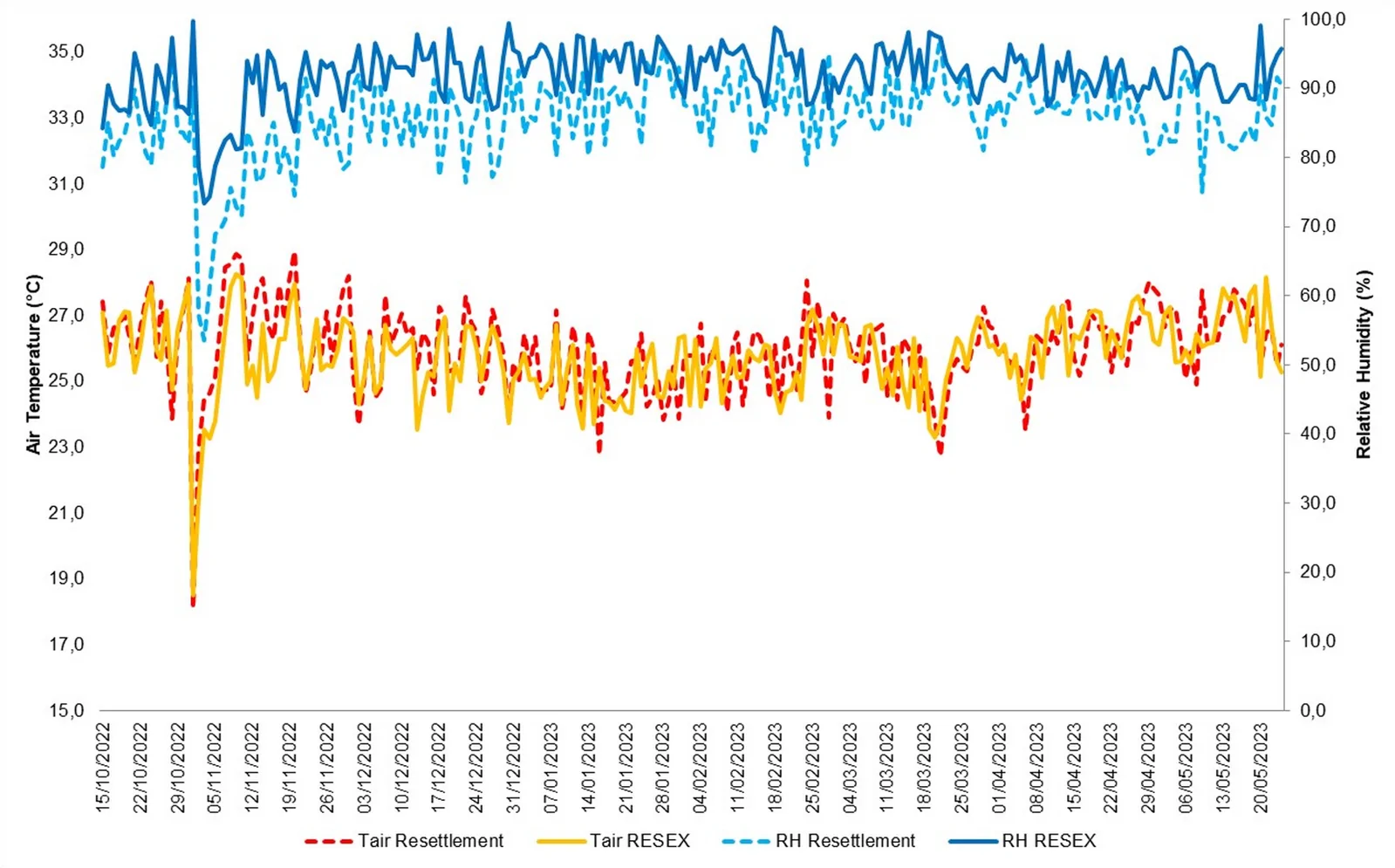
Temperature and relative humidity in RESEX and resettlement. (Source: The authors)

Table 2 presents the statistical summary for all outdoor climate variables at the two locations analyzed. Figure 8 graphically shows the average values for temperature and relative humidity in the RESEX and the Resettlement. It can be observed that the air temperature is higher in the Resettlement when compared to RESEX *(p-value: 0.00*) and the relative humidity is higher in RESEX (*p-value*: *0.00*), reflecting Lago do Cuniã and the higher concentration of vegetation.

**Table 2:** Comparison statistics of outdoor climate variables that evaluated air temperature (Tair), relative humidity (RH), rainfall, radiation (Rad) and wind, between the resettlement and the RESEX. Source: The authors

Variables	A > B	A = B	A-B	Statistics (T)	*p*-value
Tair resettlement × tair RESEX	58	0.06	0.22	8.20	0.00
RH Resettlement × RH RESEX	14	0.02	− 6.21	− 55.07	0.00
Rainfall RESEX × rainfall resettlement	11	77.82	− 0.31	− 9.62	0.00
Rad RESEX × rad resettlement	32	44.01	16.41	8.04	0.00
Wind RESEX × wind resettlement	4	0.49	− 0.71	− 107.58	0.00

**Fig. 9:**
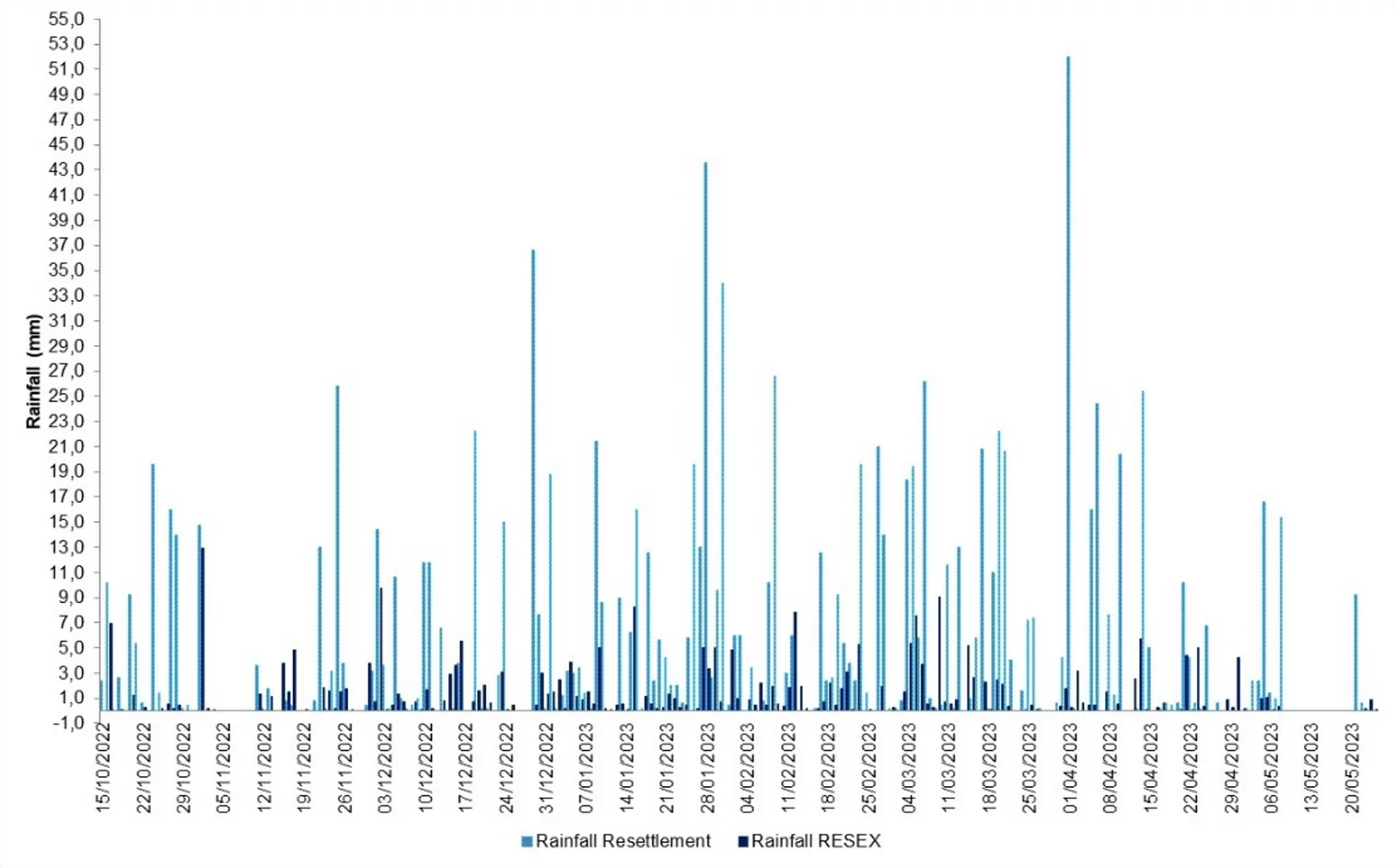
Rainfall in RESEX and the resettlement. (Source: The authors)

**Fig. 10:**
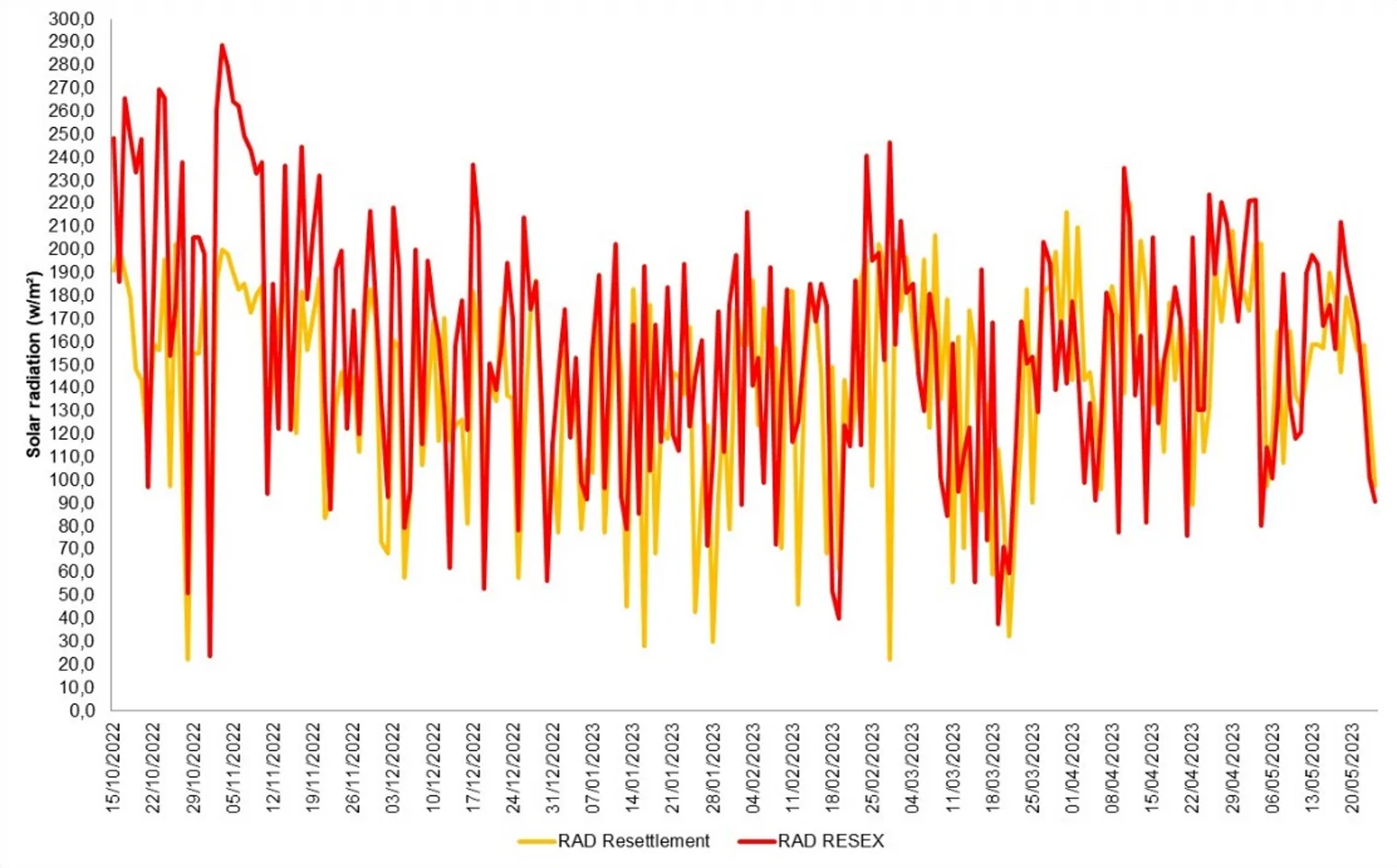
Solar radiation in RESEX and the Resettlement. (Source: The authors)

Figure 9 shows precipitation data for the two study locations, with higher rainfall totals for the Resettlement (p-value: 0.00). It can also be seen that seasonality is more pronounced in the Resettlement. Figure 8 shows the average solar radiation data, where higher values were observed in the RESEX (p-value: 0.00). Figure 10 shows the average solar radiation data, where higher values were observed in the RESEX (p-value: 0.00).

**Fig. 11:**
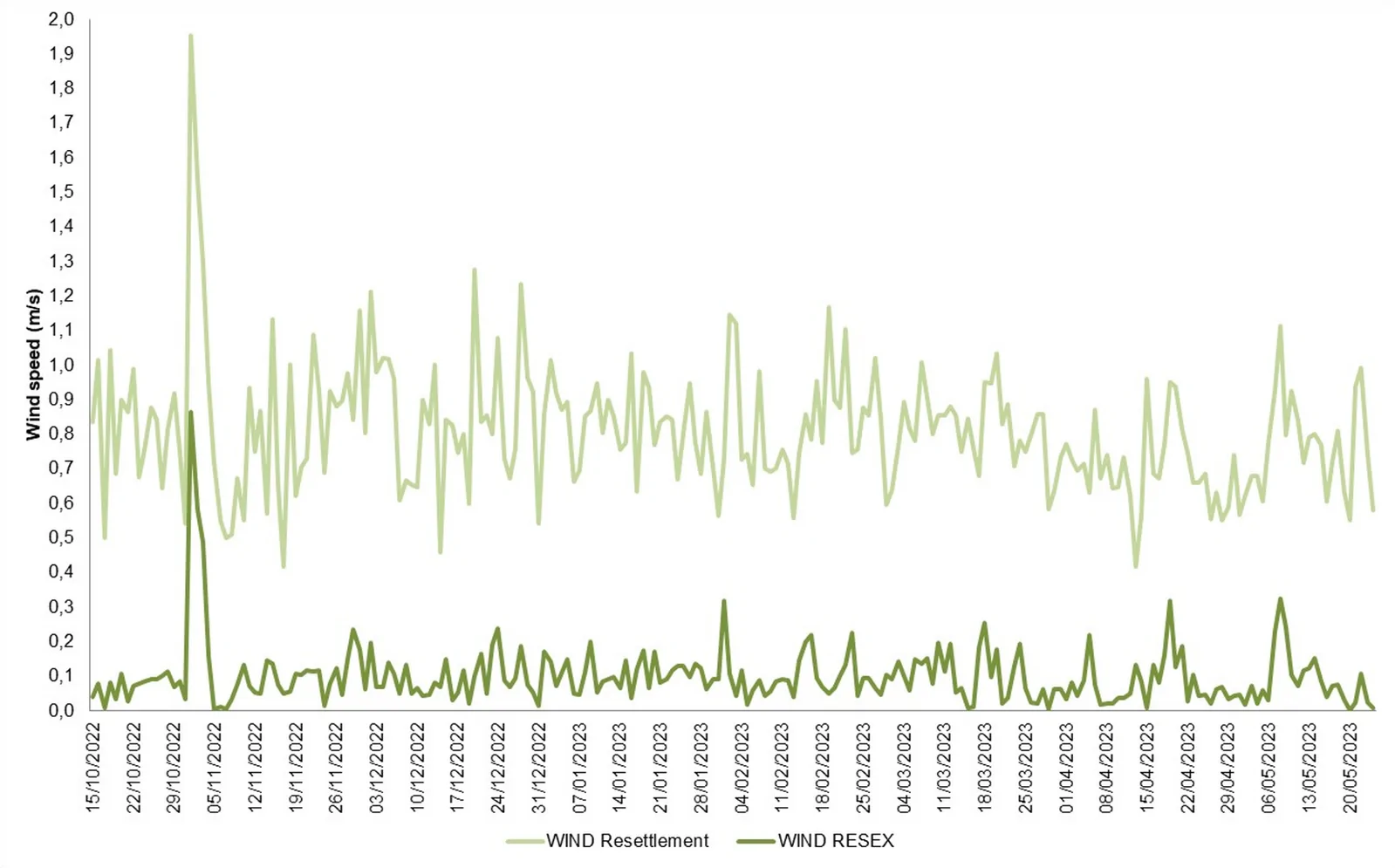
Wind speed in RESEX and the Resettlement. (Source: The authors)

Figure 11 shows the average wind speed values at the two collection points. It can be observed that the wind speed is significantly higher in the Resettlement than in the RESEX (*p-value*: 0.00).

### Normalized Difference Vegetation Index (NDVI) and Land Surface Temperature (LST)

The study period observed for the analysis of vegetation behavior in relation to the local climate was from August 2022 to August 2024. As mentioned earlier in the research method, due to local climatic conditions that include a well-established rainy season with heavy cloud cover in the region, and considering that Landsat 8 and 9 have a 16-day review period, the images showed heavy cloud cover in some months, making it impossible to produce maps representing all months of the year.

Thus, the analyses comprise the following months within the study series:

- 2022: August and September;
- 2023: May, June, July, August, September and October;
- 2024: June, July and August.

For a better understanding of the vegetation analysis within the study period, the analysis using NDVI is presented in item 3.2.1 and the analysis using LST is presented in item 3.2.2 below for both locations.

#### NDVI: Lago do Cuniã Extractive Reserve and São Domingos Resettlement

**Fig. 12:**
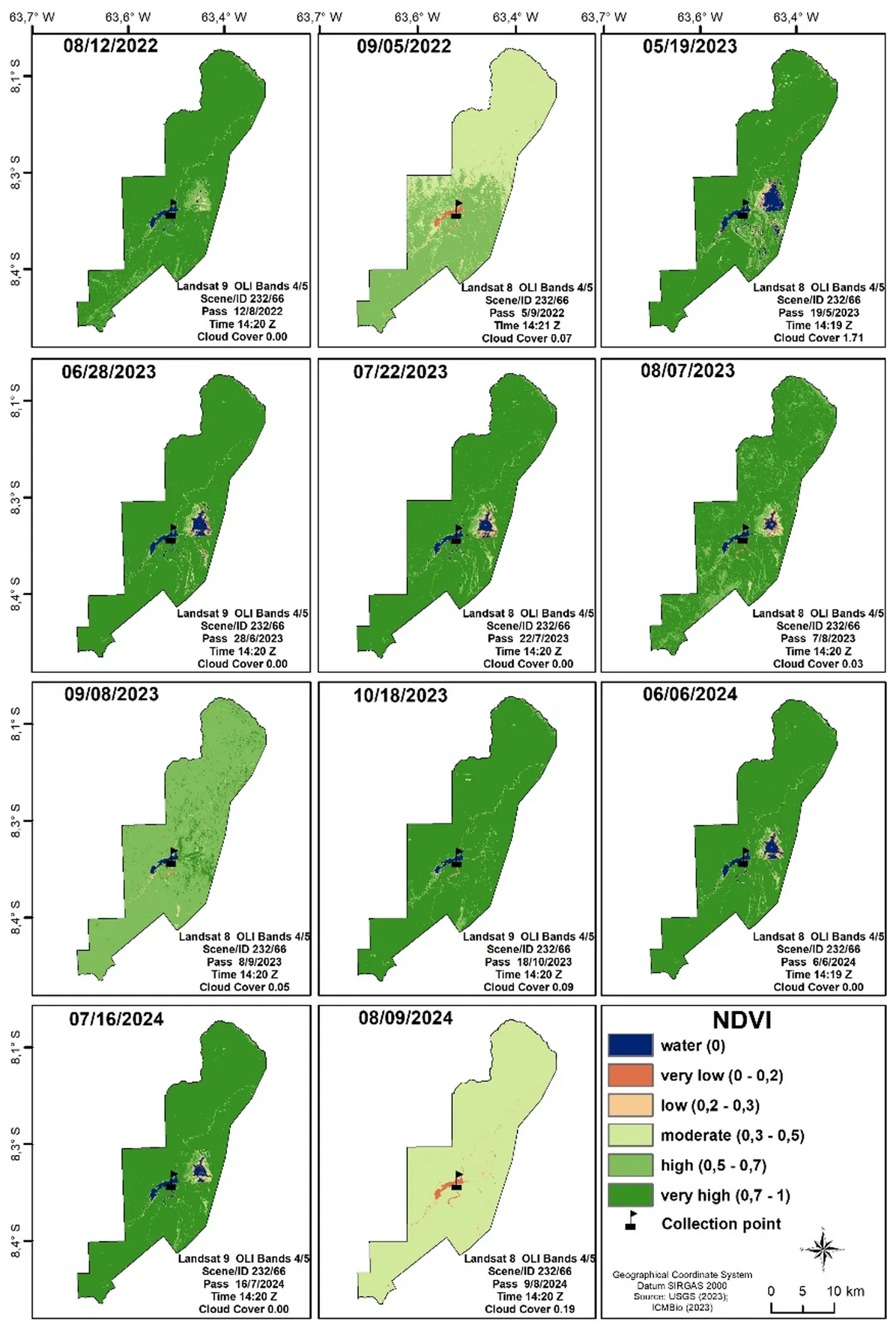
Spatial distribution of NDVI in RESEX (created using ArcMap 10.5 software: https://www.arcgis.com/index.html). (Source: The authors)

**Fig. 13:**
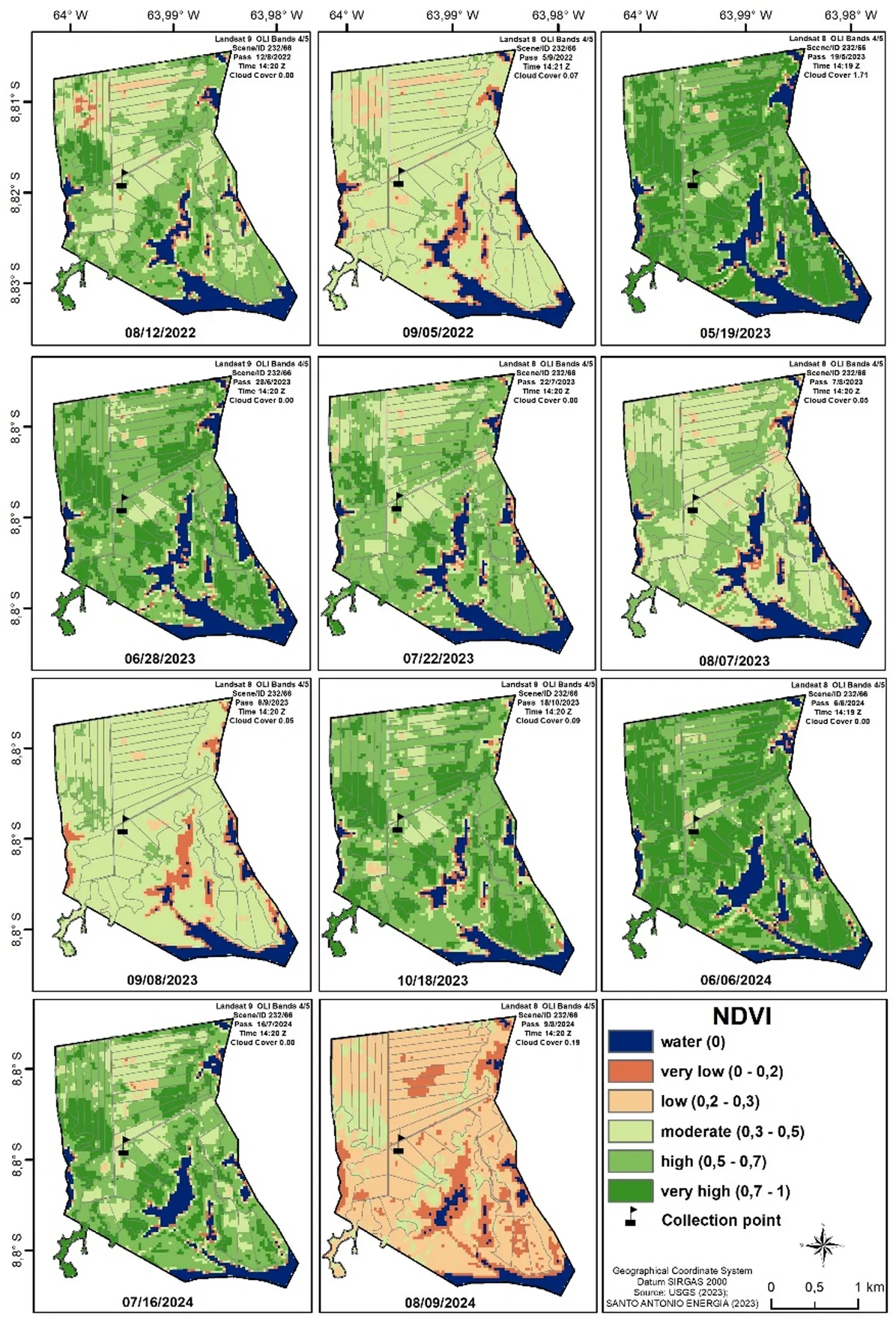
Spatial distribution of NDVI in the Resettlement (created using ArcMap 10.5 software: https://www.arcgis.com/index.html). (Source: The authors)

Figures 12 and 13 show maps with the spatial distribution of NDVI for the Lago do Cuniã Extractive Reserve and the São Domingos Resettlement, respectively.

In RESEX (Fig. 12), it can be observed that, in *2022*, from August to September, there is a decrease in vegetation areas in the “very high” class and an increase in “moderate” and “high” vegetation areas, reflecting the month of September, which represents the beginning of the transition period from the dry season to the rainy season. In *2023*, there is little monthly change within the adopted scale, with the exception of September, the beginning of the transition period from dry to rainy season, where there is a decrease in “very high” class vegetation and an increase in “high” class vegetation. In June, July, and August *2024*, there is a significant decrease in the “very high” vegetation class, as well as a significant increase in the “moderate” vegetation class in August. In addition, there is also a significant decrease in water bodies for the month of August.

In the Resettlement (Fig. 13), in 2022, there is a predominance of “moderate” and “high” class vegetation, and for the month of September, the “moderate” class predominates over the others. In *2023*, for the months of May and June, the predominant classes are “very high” and “high” vegetation. In June and July, there is a decrease in the “very high” class, with a predominance of the “high” and “moderate” classes, with an increase in the “moderate” class for the month of September. In October there is a significant increase in the “very high” and “high” classes, reflecting the beginning of the rainy season. In *2024*, the “very high” and “high” classes predominate in June and July. In August, there are no areas in the “very high” and “high” classes, with a significant predominance of “low” class vegetation.

Table 3 shows the percentage of each thematic class, adopted here, for the study series (August 2022 to August 2024) for NDVI in RESEX and the Resettlement. It can be seen that for RESEX, the predominant vegetation classes were “very high” and “high,” except for the month of August 2024, where the “moderate” class predominated. In the Resettlement, it can be observed that the “moderate” and “high” classes predominate, followed by the “very high” class, with the exception of August 2024, when the “low” class was quite significant.

**Table 3:**
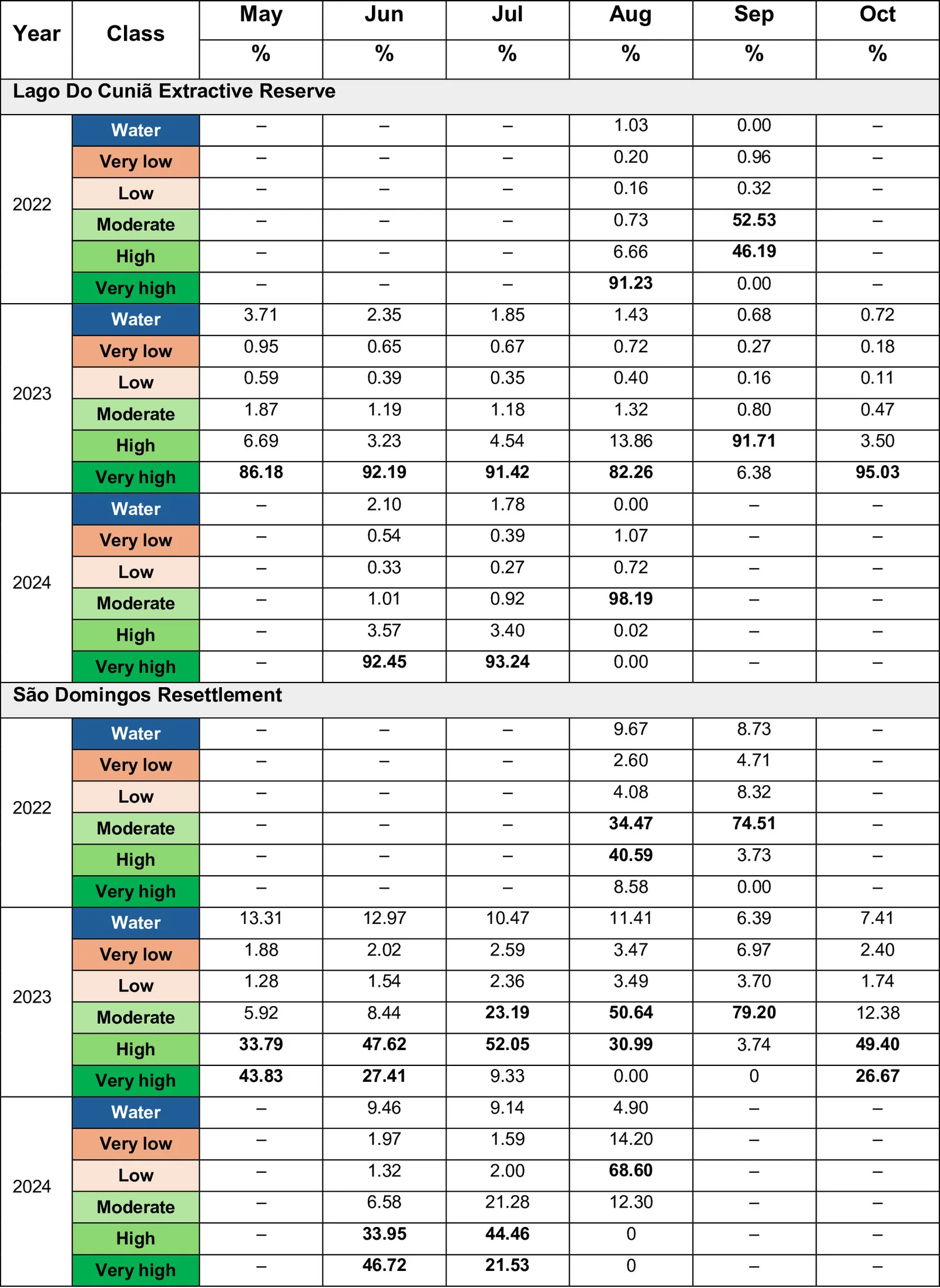
Percentage of thematic classes for NDVI in RESEX and the Resettlement. Source: The authors.

#### LST: Lago do Cuniã Extractive Reserve and São Domingos Resettlement

**Fig. 14:**
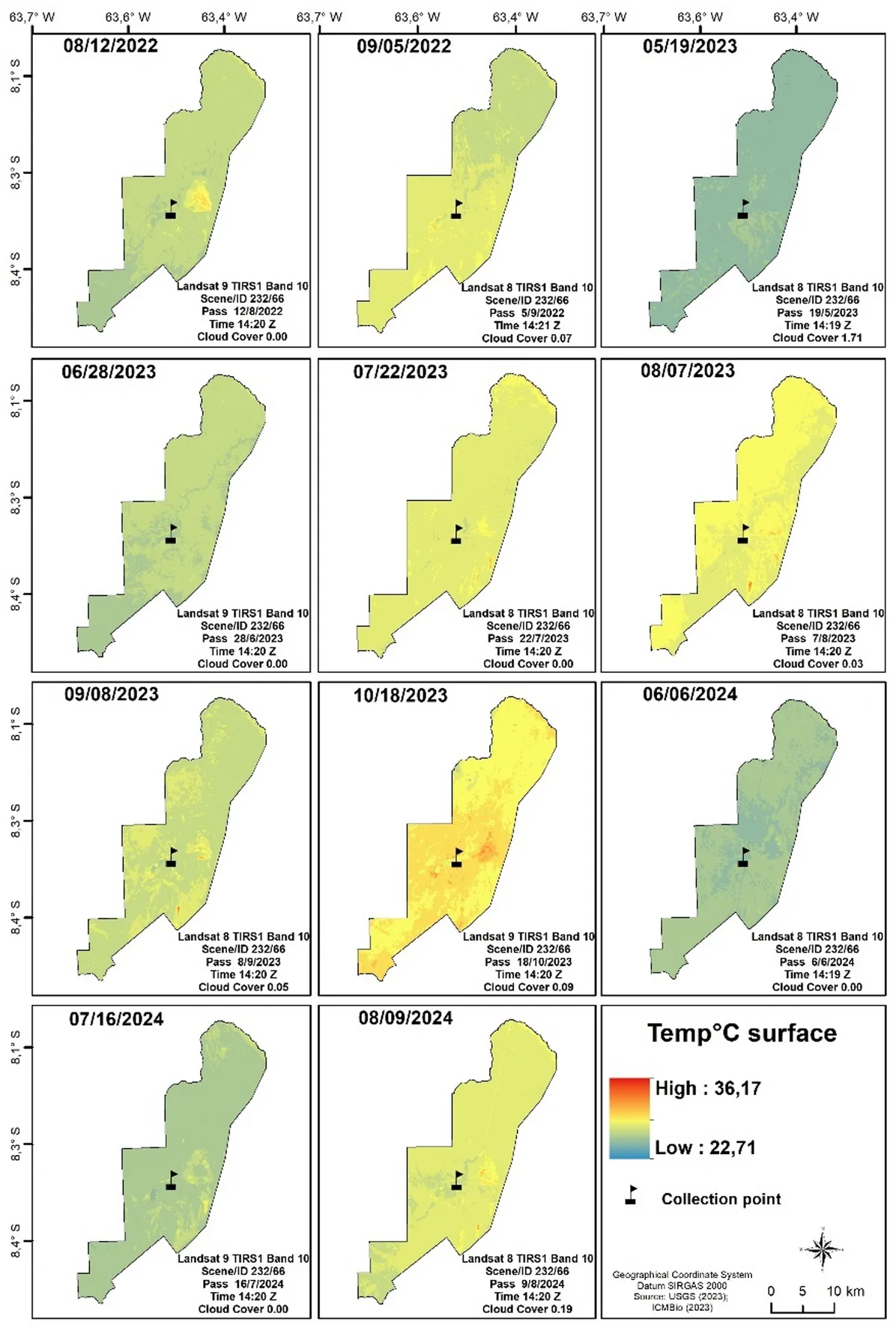
Spatial distribution of LST in RESEX (created using ArcMap 10.5 software: https://www.arcgis.com/index.html). (Source: The authors)

**Fig. 15:**
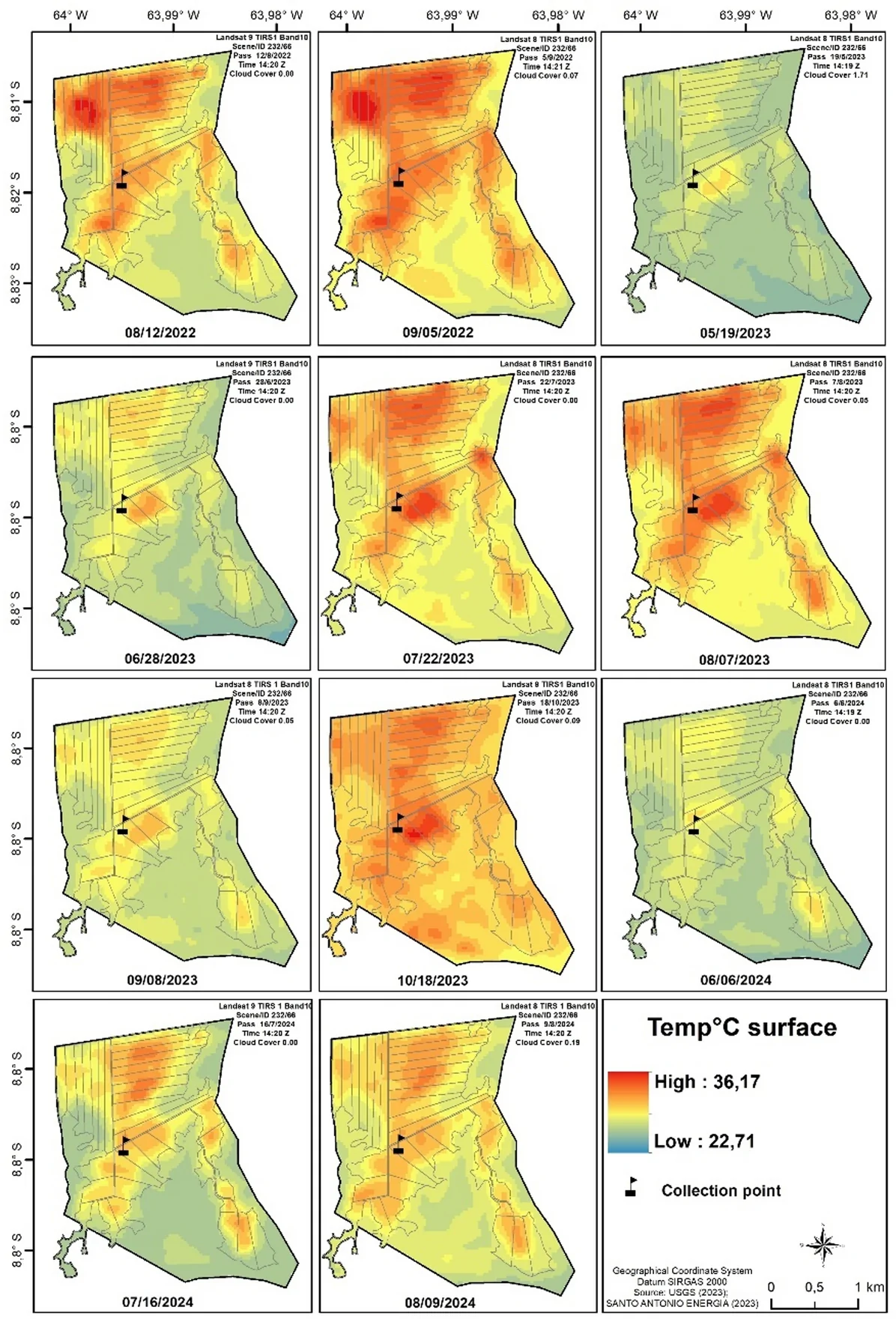
Spatial distribution of LST in the resettlement (created using ArcMap 10.5 software: https://www.arcgis.com/index.html). (Source: The authors)

Figures 14 and 15 show maps with the spatial distribution of LST for the Lago do Cuniã Extractive Reserve and the São Domingos Resettlement, respectively.

In *2022*, RESEX (Fig. 14; Table 4) shows temperatures ranging from 26 to 31 °C for the month of August, with 76.71% of the area measuring 27 °C at the target. In September, LST values range from 27 to 31 °C, with 59.63% of the area measuring 28 °C at the target. This variation between months reflects the end of the dry season in September. In *2023*, there is a temperature range of 24 °C to 34 °C between May and October, with May being the month with the lowest LST value per area (24 °C) and October with 95.2% of the area measuring between 29 and 30 °C. In *2024*, the LST ranges from 25 to 32 °C, with 77% of the area in the 26 °C range in June and 84.45% of the area in the 28 °C range in August.

**Table 4:**
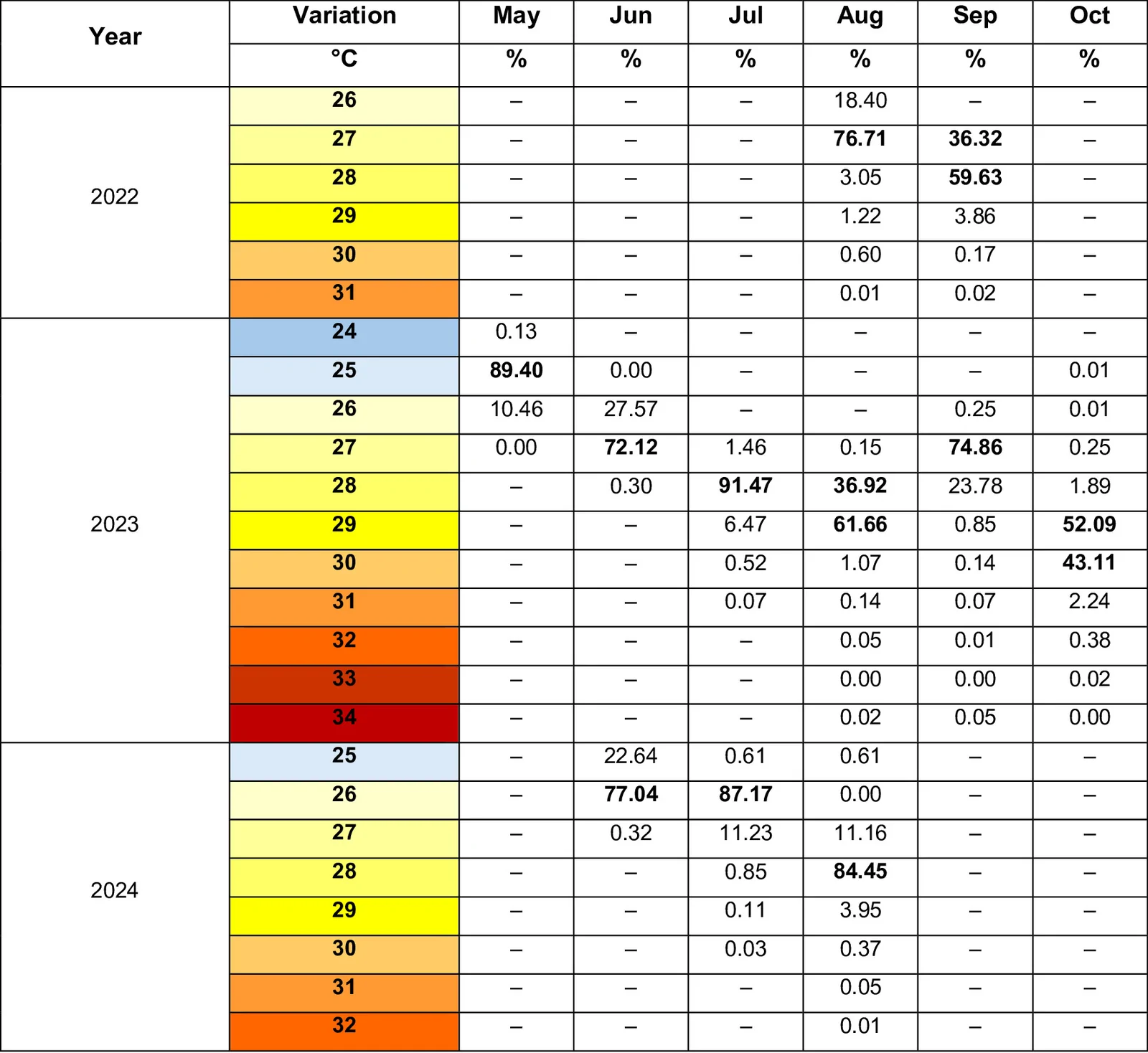
Percentage of LST variation for RESEX. Source: The authors.

In the Resettlement (Fig. 15; Table 5), in *2022*, temperatures ranging from 26 to 36 °C are observed for the month of August, with 46.65% of the area measuring between 27 °C and 28 °C at the target. In September, LST values range from 27 to 36 °C, with 41.88% of the area measuring between 29 and 30 °C at the target. In *2023*, values ranging from 24 to 36 °C are observed in the months of May to October, with an interval of 24 °C to 36 °C. May had the lowest LST values, with 49.31% of the area measuring 26 °C, and October had the highest LST values, with 64.41% of the area measuring between 30 and 31 °C. In *2024*, LST values ranged from 25 to 34 °C between June and August, with 41.58% of the area measuring 27 °C in August and 32.89% of the area measuring 28 °C in August.

**Table 5:**
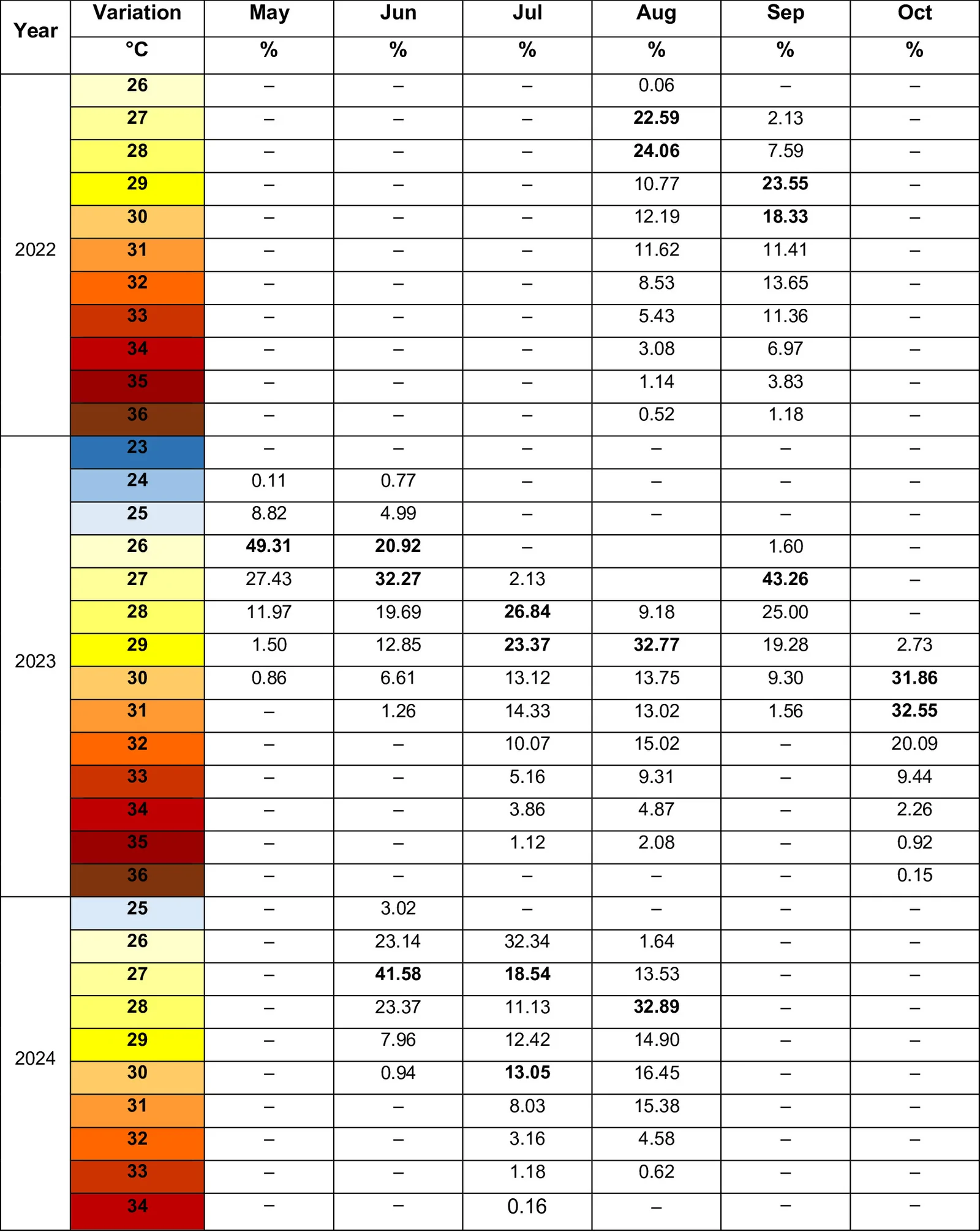
Percentage of LST variation for the Resettlement. Source: The authors

### Construction aspects and comfort of local dwellings

The Lago do Cuniã Extractive Reserve (RESEX) has dwellings of various types, including wooden houses, both stilt houses and non-stilt houses, masonry houses, and mixed houses. In the São Domingos Resettlement, as it is a community of resettled families, there are standard dwellings provided to families who lived in the flood area of the Santo Antônio Hydroelectric Plant, all of which are masonry.

In view of this, the construction types that were analyzed in both locations are presented, as well as an analysis of their comfort in the context of the adaptive comfort model.

#### Dwellings and the adaptive comfort model

**Fig. 16:**
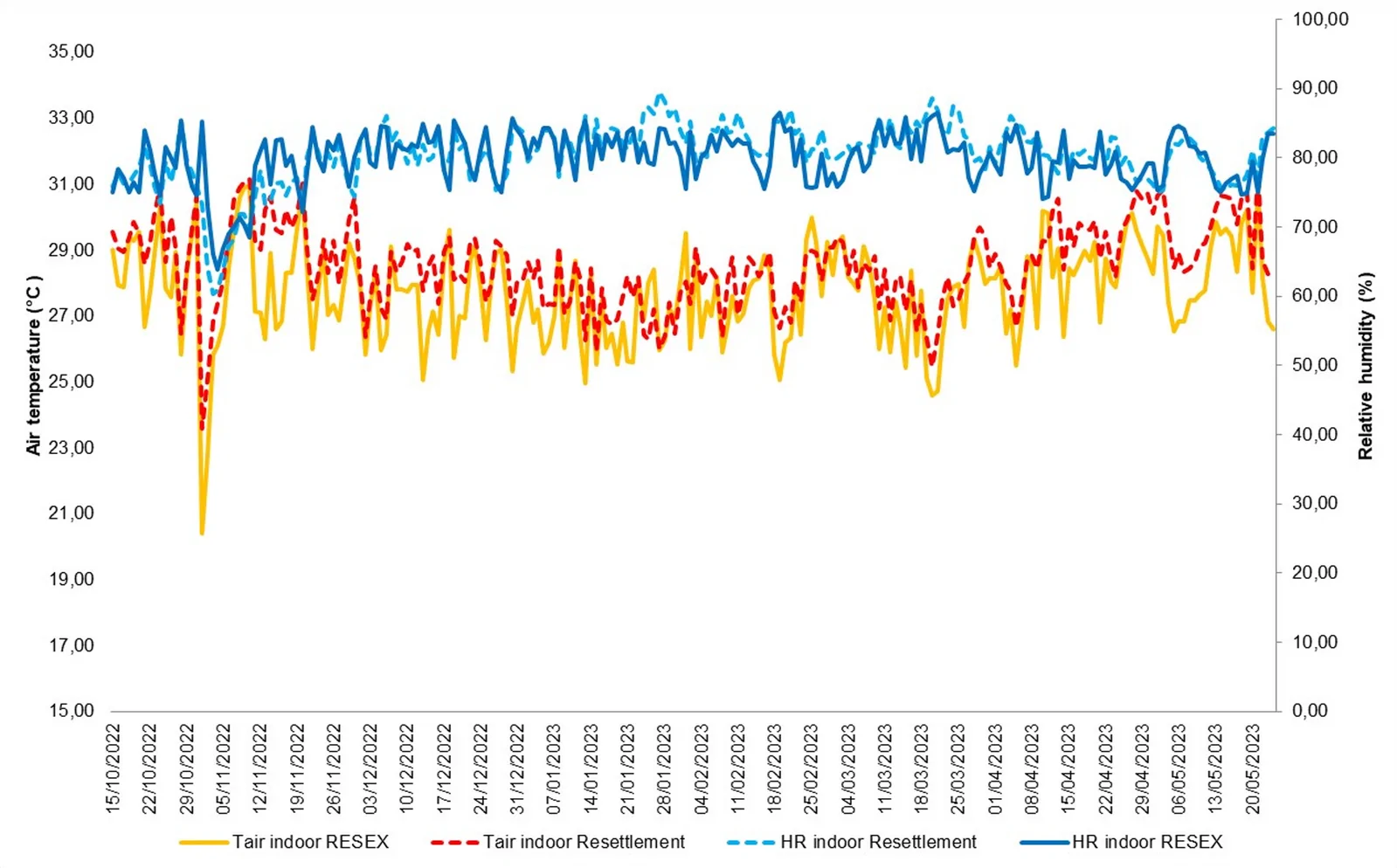
Indoor temperature and relative humidity in RESEX and the Resettlement. (Source: The authors)

Before beginning the analysis of the data collected using the Adaptive Comfort Model, it is important to observe the data collected inside the dwellings. Figure 16 shows the average values for indoor temperature and relative humidity, and Table 6 presents a statistical summary of the indoor microclimatic variables in RESEX and the Resettlement, comparing them with the outdoor data.

The data show that, although graphically there is a slight difference between the two dwellings, statistically, the indoor temperature is higher in the Resettlement *(p-value: 0.00*), as shown in Table 6. It is also observed that the indoor temperature data are higher than the outdoor temperature data *(p-value: 0.00*), both in RESEX and in the Resettlement (Table 6).

The indoor relative humidity is different in the two dwellings (*p-value 0.00*), with higher values observed in the Resettlement dwelling. When indoor relative humidity is compared to outdoor relative humidity, it is observed that indoor relative humidity is lower in RESEX and the Resettlement *(p-value: 0.00*), as shown in Table 6.

**Table 6:** Statistics of climatic (outdoor) and microclimatic (indoor) variables that pointed to differences between the dwellings analyzed. Source: The authors

Variables	A > B	A = B	A-B	Statistics (T)	*p*-value
Tair indoor resex × tair indoor resettlement	28	0.77	− 0.83	− 23.31	0.00
Tair indoor resex × tair outdoor resex	85	0.04	1.96	54.82	0.00
Tair indoor resettlement × tair outdoor resettlement	91	0.49	2.57	105.27	0.00
RH indoor resex × RH indoor resettlement	50	0.98	− 0.54	− 7.13	0.00
RH indoor resex × RH outdoor resex	8	0.02	− 11.99	− 124.89	0.00
RH indoor resettlement × RH outdoor resettlement	25	0.26	− 5.24	− 41.26	0.00

The data presented make the following comparisons for Air Temperature (Tair) and Relative Humidity (RH): indoor data between RESEX and the Resettlement; outdoor and indoor data in RESEX and outdoor and indoor data in the Resettlement.

Given the data presented, it could be inferred that the dwellings in the Resettlement are warmer and more humid than the dwellings analyzed in RESEX. However, to verify the comfort level of the occupants in these dwellings, the Adaptive Comfort Model for Southeast Asia was used. The comfort range with 80% acceptance is approximately ± 3 °C around the comfort temperature indicated by the model^34^, and the result of its application is presented in Table 7. It can be observed that the comfort temperature obtained by the model varied by 0.5 °C during the period analyzed, presenting the same interval (27.4 °C and 27.9 °C) for both study locations.

**Fig. 17:**
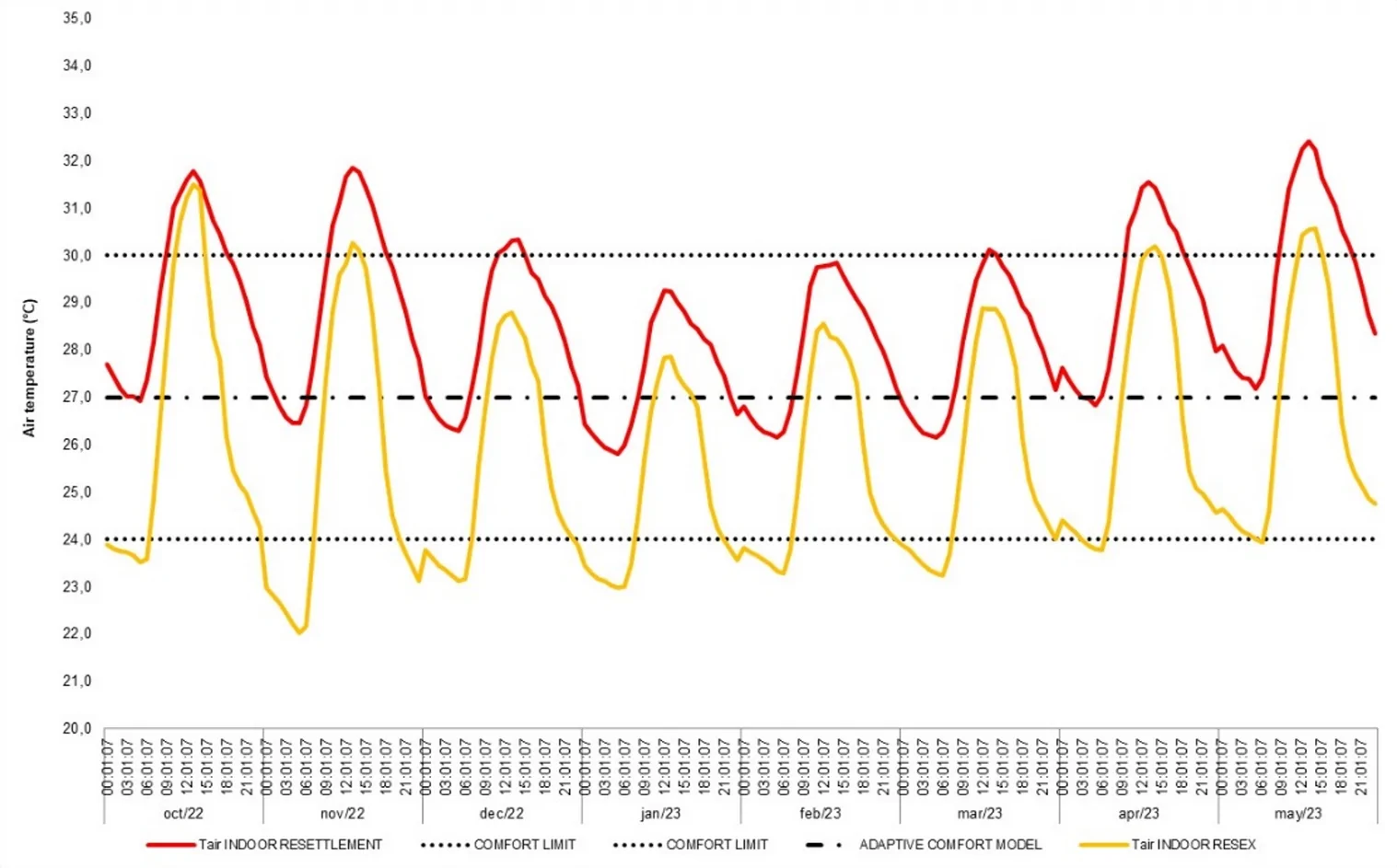
Dwellings analyzed and the adaptive comfort model. (Source: The authors)

Figure 17 graphically represents the thermal behavior of the dwellings observed in relation to the adaptive comfort model for the entire analysis period. As the variation in comfort temperature, presented in Table 7, was quite discreet between the study locations, it was possible to construct a graph with information from both dwellings, which allows for better observation of their thermal behavior.

**Table 7:** Average outdoor temperatures, monthly comfort temperature according to the adaptive comfort model, and comfort temperature range with 80% acceptance for RESEX and the resettlement. Source: The authors

Data source	Month/year	Average outdoor temperature (°C)	Comfort temperature—adaptive comfort model (°C)	Comfort temperature range (°C)—80% acceptance
RESEX				
Campbell CR1000 Weather Station	Oct/2022	26.5	27.9	24.9 to 30.9
	Nov/2022	25.5	27.5	24.5 to 30.5
	Dec/2022	25.6	27.5	24.5 to 30.5
	Jan/2023	25.0	27.4	24.4 to 30.4
	Feb/2023	25.5	27.5	24.5 to 30.5
	Mar/2023	25.6	27.6	24.6 to 30.6
	Apr/2023	26.3	27.8	24.8 to 30.8
	May/2023	26.6	27.9	24.9 to 30.9
Resettlement				
Santo Antônio Hydroelectric Power Plant Weather Station	Oct/2022	26.6	27.9	24.9 to 30.9
	Nov/2022	26.4	27.8	24.8 to 30.8
	Dec/2022	25.9	27.6	24.6 to 30.6
	Jan/2023	25.1	27.4	24.4 to 30.4
	Feb/2023	25.7	27.6	24.6 to 30.6
	Mar/2023	25.5	27.5	24.5 to 30.5
	Apr/2023	26.1	27.7	24.7 to 30.7
	May/2023	26.6	27.9	24.9 to 30.9

Given the comfort temperature range, which varied between 24.4 and 30.9 °C, it was observed that the dwelling located in RESEX, the region furthest from the urban area of Porto Velho, which is constructed of wood and has an unlined roof in the environment where the data were collected, presented average air temperature values ranging from 22.0 to 31.4 °C, remaining within the model’s comfort range for most of the period, with brief periods below the lower limit of the acceptance range. According to Nguyen et al.^28^, occupants tend to have better acceptance for intervals below the lower limit of the comfort range than above the limit established by the model.

The dwelling located in the Resettlement, a region close to the urban area of the capital of Rondônia, built in masonry and with PVC ceiling panels in the data collection environment, presented, for the study period, average air temperature values that varied between 25.8 and 32.4 °C, also remaining within the comfort range for most of the period, however, it presented air temperature peaks above the upper comfort range limit in the months of October and November 2022, as well as April and May 2023.

Thus, observing the microclimatic data that proved to be statistically different in the two dwellings analyzed and considering Fig. 17, relating to the average thermal behavior of the dwellings within the observed period, it can be inferred that the dwelling analyzed in the RESEX is more comfortable for the occupants than the dwelling analyzed in the Resettlement, given the variables considered.

## Living in the Amazon: critical analysis and discussion of results

To investigate “living in the Amazon,” as proposed in this research, in two riverside communities in the capital of Rondônia, local climate variables, local vegetation, and riverside architecture were used in the study contexts. In this scenario, two local communities are analyzed: the São Domingos Resettlement, near the urban perimeter of the city, which has masonry dwellings with a standard design developed by the Santo Antônio Hydroelectric Plant for the location, and the Lago do Cuniã Extractive Reserve, far from the urban perimeter, which has traditional dwellings in the region using wood as the main raw material. With these data, it was possible to understand how riverside architecture, traditional or not, behaves within areas of lesser and greater environmental conservation.

When analyzing the climatic variables in the study locations, it was possible to perceive differences between both for all the variables observed. The local climate is strongly influenced by the regional climate^35–38^, and the different microclimates of the Amazon region are directly influenced by the local climate^36^. considers 15 to 150 km to be an appropriate spatial scale for the local climate. However, even though the two collection points observed here (RESEX and Resettlement), which are approximately 80 km apart, are within the scale considered by Ribeiro^36^, the data collected here showed the influence of the urban perimeter of Porto Velho on the microclimate of the São Domingos Resettlement.

This influence can be observed when analyzing the air temperature, which was higher in the Resettlement Area, which seems to be related to its proximity to the capital’s urban perimeter. Relative humidity was higher in RESEX. This reflects environmental conditions, where the body of water of Lake Cuniã (near the weather station that collected the data) and dense vegetation result in higher relative humidity values^39,40^ and, consequently, lower air temperature values.

As for precipitation, maximum average values of 52.0 mm were observed for the Resettlement, compared to 13.0 mm in the RESEX, meaning that during the period observed, it rained considerably more in the Resettlement. This data seems to reflect the possible impact of the urban heat island of the Porto Velho urban perimeter, which in turn can generate low atmospheric pressure centers, promoting thermal convection and the development of deeper rain clouds^41,42^, especially during the rainy season observed. This higher concentration of rain clouds in the Resettlement was reflected in lower solar radiation values for that location, compared to RESEX, since cloud cover acts as a mechanism to block the entry of radiation.

The Resettlement presented higher values for wind speed, with maximum averages of 2.0 m/s, while in RESEX, 0.9 m/s was observed. In this data, the influence of vegetation acting as a wind blocking mechanism can be observed^43^.

NDVI was used to assess the distribution of vegetation in the study regions. Due to cloud cover during the rainy season and considering that precipitation ends up saturating NDVI values, making it difficult to separate vegetation classes during this period^44^, images from the dry/transition periods were used within the adopted series, since precipitation is directly related to photosynthetic activity^45^.

In RESEX, the maps show small variations between the study period (August 2022 to August 2024), obviously respecting the limitations of remote sensing research, considering that it was not possible to produce maps for all months of the series.

That said, it can be seen that, for the study series, the presence of “very high” class vegetation predominates in RESEX in all years of the study. In August and September 2022, a difference was observed that reflects the transition between the dry and rainy seasons, which was also observed in the same period in 2023, with an increase in the areas of “high” and “moderate” vegetation classes in August. However, while in August 2022 and 2023 the maps show that the vegetation was predominantly in the “very high” class with values of 91% and 82%, respectively, in August 2024, 98% of the area was observed to be in the “moderate” vegetation class, i.e., a drop of two levels within the class adopted in the NDVI maps (six classes). These data represent a considerable reduction in water storage in the system, since 2024 was the driest year on record for the Brazilian Amazon.

Based on^46^, in 2023/2024 there were extreme droughts in the Brazilian Amazon, and during the rainy season of these years, rainfall was below normal, especially in the western Amazon, which prevented substantial water recharge for the climate system. Consequently, the dry season of 2024 was the driest for the region, which can be observed by the gradual increase in land surface temperature, as seen in the LST maps developed for this study.

The NDVI maps developed for the Resettlement show that, during the study period, the “very high,” “high,” and “moderate” classes predominated, unlike what was observed in the RESEX. When looking at August 2022 and August 2023, 75% and 81% of the area was concentrated in the “high” and “moderate” classes, respectively. In August 2024, the “moderate” class predominated in 69% of the study area. However, unlike the data observed in the RESEX maps, the Resettlement had its most critical period in September 2022 and 2023, with 74% and 79%, respectively, of the vegetation in the “moderate” class.

Thus, when analyzing the two study areas, it is clear that RESEX has a greater potential for vegetation regeneration than the Resettlement, since the month following the most critical period in the Reserve, within the adopted scale, shows rapid vegetation regeneration, while in the Resettlement there is a lower capacity for this regeneration with the extension of the critical period.

Given the climatic and vegetative data, the objective was to evaluate the thermal behavior of the local architecture. Statistical analysis showed that indoor temperature and relative humidity data differ between the riverside communities analyzed, with higher values observed in the Resettlement dwelling, with a difference of 3.8 °C for minimum average values and 1.0 °C for maximum average air temperature values, for example.

However, given that the riverside architecture analyzed has different construction standards, from the perspective of construction materials and techniques, both traditional and contemporary, outdoor air temperature data was used to estimate the indoor comfort temperature of each dwelling and thus to evaluate the thermal behavior of the riverside architecture in question. The Adaptive Comfort Model was used, which considers the theory of adaptive comfort that contemplates, among other factors, the adaptive attitudes of occupants to promote better comfort conditions, such as window control, variation in activity levels, and clothing changes^28^.

Thus, based on the monthly average outdoor temperature, it was possible to obtain the estimated comfort temperature for the two dwellings, which showed a slight variation in the study period, between 27.4 and 27.9 °C, and the comfort temperature acceptability range with 80% acceptance.

The data obtained show that both dwellings analyzed remained within the comfort temperature acceptability range for most of the period analyzed, observing the average indoor temperature values. The riverside dwelling studied in the RESEX presented temperature intervals that varied for brief periods above the upper limit and below the lower limit of the comfort range. The dwelling studied in the Resettlement showed brief periods above the upper limit.

According to data collected by^28^, “occupants are less sensitive to thermal change on the colder side of the comfort temperature range and vice versa.” Based on this and considering the statistical analysis of the internal data, it is possible to state, for the dwellings that were the subject of our study, that the traditionally built riverside dwelling, using wood as its main raw material, seems to offer better comfort conditions to occupants when compared to the masonry dwelling in the Resettlement. This finding encourages the continuation of traditional riverside architecture, the use of wood as a raw material, and the perpetuation of traditional knowledge, which has produced Amazonian riverside architecture for decades and adapts its existence to the forest.

The results presented here show the important role of vegetation conservation, as a greater capacity for regeneration of the green area was observed, due to its density in the RESEX, which in turn seems to influence the thermal comfort of local dwellings. In addition, the vegetation density near the Lago do Cuniã water body seems to positively influence the local microclimate.

The present research was unable to evaluate or estimate how the thermal behavior of the dwellings analyzed here, one traditional in wood and the other contemporary in masonry, would be if they were in opposite environments, for example. However, the observed data show that the binomial “traditional architectural techniques”, which carry traditional riverside knowledge, and “vegetation conservation” seems to favor thermal comfort in riverside communities.

## Final considerations

With the aim of understanding the dynamics of “living in the Amazon,” in two riverside communities, in areas of greater and lesser environmental conservation, this research proposed an interdisciplinary analysis and discussion on vegetation conservation, local climate, and thermal comfort in the traditional architecture of riverside communities in the Brazilian Amazon, based on field measurements, remote sensing techniques, and architectural analysis.

The results obtained through the adopted method showed differences in the local climate in both locations. Based on the data collected, it was possible to see that the conservation of vegetation impacted the local climate. In addition, through the NDVI and LST maps, it was observed that the higher density of vegetation in the RESEX reflected in the greater capacity for regeneration of this vegetation after critical periods, such as the dry period of 2023/2024.

Considering that dwellings of different construction types were analyzed in different contexts, it can be inferred that traditional riverside architecture, together with the context of greater environmental conservation in the RESEX, provides better comfort conditions for occupants, encouraging the production of vernacular architecture.

However, the study has limitations and gaps that can be clarified in future research:

- It was not possible to analyze dwellings of the same type in the opposite context to that found in the present study;
- It was not possible to produce NDVI and LST maps for all months of the study series.

Nevertheless, despite the limitations presented, the data observed here are consistent and robust enough to affirm that vegetation conservation plays an important role in the local climate, as well as in the comfort of traditional riverside dwellings. In addition, the results presented here serve as a contribution for public authorities to act more precisely in environmental conservation and in the disorderly and unplanned advancement of the urban perimeter of the capital of Rondônia.

## Data Availability

The data supporting the conclusions of this research are available to readers upon request to the corresponding author.

## References

[1] Pereira, C. A. R., Winkler, M. S. & Hacon, S. S. Análise de Condições Ambientais em Comunidades Ribeirinhas de Porto Velho, Rondônia, Brasil. *Revista Brasileira de Geografia Física*. 10.26848/rbgf.v9.2.p440-455 (2016).

[2] Nogueira, R. L. B. Arquitetura Vernacular e Paisagem Amazônica: um Caminho na Busca pelo Habitar Poético. *Revista da Abordagem Gestáltica—Phenomenological Studies*. **22**(2), 171–180 (2016).

[3] Mazzone, A. Thermal comfort and cooling strategies in the Brazilian Amazon. An assessment of the concept of fuel poverty in tropical climates. *Energy Policy*. 10.1016/j.enpol.2020.111256 (2020).

[4] Celuppi, M. C. Arquitetura e percepçăo bioclimática em habitaçőes ribeirinhas na Amazônia Brasileira. *Dissertação* (Mestrado em Arquitetura e Urbanismo) Universidade Presbiteriana Mackenzie, 199 p. (2018).

[5] Celuppi, M. C., Meirelles, C. R. M., Cymrot, R., Borst, B. A. & Gobo, J. P. A. Preliminary approach to the analysis of climate perception and human thermal comfort for riverside dwellings in the Brazilian Amazon. *J. Building Eng.* **23**, 77–89 (2019).

[6] Sampaio, M. R. A. & Lencione, S. *Casas do Brasil: Habitação Ribeirinha na Amazônia* (Museu da Casa Brasileira, 2013).

[7] Takano, T., Nakamura, K. & Watanabe, M. Urban residential environments and senior citizens’ longevity in megacity areas: the importance of walkable green spaces. *J. Epidemiol. Community Health*. **56**, 913–918. 10.1136/jech.56.12.913 (2002). 12461111 PMC1756988

[8] Lovasi, G. S., Quinn, J. W., Neckerman, K. M., Perzanowski, M. S. & Rundle, A. Children living in areas with more street trees have lower prevalence of asthma. *J. Epidemiol. Community Health*. **62**, 569–569. 10.1136/jech.2007.071894 (2008). PMC3415223 18450765

[9] BERG, A. V., MAAS, J., Verheij, R. A., & GROENEWEGEN, P. P. Green space as a buffer between stressful life events and health. *Soc. Sci. Med.* **70**, 1203–1210 (2010). 20163905 10.1016/j.socscimed.2010.01.002

[10] Dobbert, L. Y., Prata-Shimomura, A. R., Zanlorenzi, H. C. P. & Franco, M. A. R. Percepção e conforto dos usuários do Parque Trianon em São Paulo/SP. *Revista LABVERDE*. 10.11606/issn.2179-2275.v8i2p59-73 (2017).

[11] Moreira, T. C. L. et al. Green spaces, land cover, street trees and hypertension in the megacity of São Paulo. *Int. J. Environ. Res. Public. Health*. **17**, 725. 10.3390/ijerph17030725 (2020). 31979152 PMC7038323

[12] Shin, J. C., Parab, K. V., An, R., & Grigsby-Toussaint, D.S. Greenspace exposure and sleep: a systematic review. *Environ. Res.* 10.1016/j.envres.2019.109081 (2020). 31891829

[13] xie, J., Luo, S., Furuya, K. & Sun, D. Urban parks as green buffers during the COVID-19 pandemic. *Sustainability*10.3390/su12176751 (2020).

[14] Pires, D. G. M. Qualidade ambiental da cidade de Porto Velho análise por meio da quantificação da cobertura vegetal e áreas verdes para o perímetro urbano. Dissertação de Mestrado. Pós-graduação em Desenvolvimento Regional e Meio Ambiente, Área de Concentração em Políticas Públicas e Desenvolvimento Sustentável. Universidade Federal de Rondônia UNIR, 156. (2019).

[15] Instituto Brasileiro de Geografia e Estatística—IBGE. (2020). https://www.ibge.gov.br/cidades-e-estados/ro/porto-velho.html

[16] Köppen W. Versuch einer Klassifikation der Klimate, vorzugweise nach ihren Beziehungen zur Pflanzenwelt. *Meteorol. Z.* **18**, 106–120 (1901).

[17] Alvares, C.A., Stape, J.L., Sentelhas, P.C., de Moraes Goncalves, J.L., & Sparovek G. Köppen’s climate classification map for Brazil. *Meteorol. Z.* **22** (6), 711–728 (2013).

[18] Novais, G. T., & Machado, L. A. Os climas do Brasil: segundo a classificação climática de Novais. *Revista Brasileira De Climatologia*. **32** (19), 1–39. 10.55761/abclima.v32i19.16163 (2023).

[19] Santos Neto, L. A., DISTRIBUIÇÃO HORÁRIA DA PRECIPITAÇÃO EM PORTO & VELHO-RO. *Revista Brasileira de Climatologia*, [S.l.], v. 14, 2014. ISSN 2237–8642 (2014).

[20] Bezerra, R. B., Dantas, R. T. & Trindade, A. G. Caracterização Temporal da Precipitação Pluvial do Município de Porto Velho/RO no Período de 1945 a 2003. *Sociedade & Natureza Uberlândia-MG* **22** (3), 609–623. 10.1590/S1982-45132010000300015 (2010).

[21] Fisch, G., Marengo, J. A. & Nobre, C. A. Uma revisão geral sobre o clima da Amazônia. *Acta Amaz*. 10.1590/1809-43921998282126 (1998).

[22] Tejas, T. G., Nunes, D. D., Souza S. M. R., Correa S. C. A. & Watanabe, M. Análise da temperatura de superfície em ambientes urbanos: um estudo por meio do sensoriamento remoto na cidade de Porto Velho/RO (1985–2011). *Confins*10.4000/confins.12191 (2017).

[23] Silva, M. J. G. et al. Cobertura do Solo e a Variabilidade da Temperatura e da Umidade Relativa do Ar em Porto Velho (RO) entre 1971–2005. In: 15º Congresso Brasileiro De Meteorologia, Belém, Anais. Belém (2010). (2010).

[24] Bastos, T. X. & Diniz, T. D. A. *S. Avaliação do clima do estado de Rondônia para o desenvolvimento agrícola* (EMBRAPA-CPATU, 1982).

[25] *ICMbio*. Plano de manejo da Reserva Extrativista Lago do Cuniã. Brasília, Disponível em: (2018). https://www.icmbio.gov.br/portal/images/stories/plano-de-manejo/plano_de_manejo_da_resex_lago_do_cunia_2018.pdf. Acesso 30 de jan. de 2022 (2018).

[26] Gil, A. C. *Métodos e técnicas de pesquisa social*. 4 ed. São Paulo: Atlas, 207 p. (1994).

[27] American Society of Heating. Refrigerating an Air-Conditioning Engineers, Inc. *ASHRAE 55*, Thermal Environmental Conditions for Human Occupancy. ASHRAE, Atlanta, GA. (2010).

[28] Nguyen, A. T., Singh, M. K. & Reiter, S. An adaptive thermal comfort model for hot humid southeast Asia. *Build. Environ.* **56**, 291–300. 10.1016/j.buildenv.2012.03.021 (2012).

[29] Toe, D. H. C., & Kubota T. Development of an adaptive thermal comfort equation for naturally ventilated buildings in hot–humid climates using ASHRAE RP-884 database. *Front. Architectural Res.* **2**, 278–291. 10.1016/j.foar.2013.06.003 (2013).

[30] Mishra, A. K. & Ramgopal, M. An adaptive thermal comfort model for the tropical climatic regions of India (Koppen climate type A). *Build. Environ.* **85**, 134–143. 10.1016/j.buildenv.2014.12.006 (2015).

[31] Mishra, A. K. & Ramgopal, M. Adaptive thermal comfort standards —the way out for burgeoning building energy needs of India. in *3rd National Conference on Refrigeration and Air Conditioning.* (2013).

[32] Lachireddig, G. K. K., Muthukumar, P. & Subudhi, S. Thermal comfort analysis of hostels in National Institute of Technology Calicut, India. *Sãdhanã* **42** (1), 63–73. 10.1007/s12046-016-0572-x (2017).

[33] Coelho, A. L. N. & de Corrêa, W. Temperatura de Superfície Celsius do Sensor TIRS/Landsat-8: metodologia e aplicações. *Revista Geográfica Acadêmica*. **7** (1), 31–45 (2013).

[34] Nguyen, A. T., Reiter, S. & Rigo, P. A review on simulation-based optimization methods applied to building performance analysis. *Appl. Energy*. **113**, 1043–1058. 10.1016/j.apenergy.2013.08.061 (2014).

[35] Monteiro, C. A. F. *Teoria e clima urbano. Série Teses e Monografias, 25* 181 (Instituto de Geografia/USP, 1976).

[36] Ribeiro, A. G. As escalas do clima. *Bol. de Geografia Teorética*. **23** (45–46), 288–294 (1993).

[37] Oke, T. R. *Initial Guidance to obtain representative meteorological observations at Urban Site, Instruments and Observing Program*. *IOM Rep.*, **81**, (2004).

[38] Gobo, J. P. A., Galvani, E. & Wollmann, C. A. Influência do clima regional sobre o clima local a partir do diagnóstico de abrangência espacial e extrapolação escalar. *Revista Brasileira de Climatologia*. **22**, 210–228. 10.5380/abclima.v22i0.57827 (2018).

[39] Yang, T. et al. Synergistic effects of vegetation greening and irrigation on summer climate in the Sichuan Basin, China. *J. Hydrology: Reg. Stud.* **61**, 102612. 10.1016/j.ejrh.2025.102612 (2025).

[40] Karuppiah, C. et al. Spatio-temporal assessment of vegetation response to climatic variability using NDVI and VCI in the forested landscape. *Rangel. Ecol. Manage.* **105**, 25–36. 10.1016/j.rama.2025.12.007 (2026).

[41] Fu, X. et al. Impacts of urban heat island on the convective initiation and propagation of an extreme precipitation event in the coastal megacity of Shanghai. *Atmos. Res.* 10.1016/j.atmosres.2025.108595 (2026).

[42] Sadra, N., Nikoo, M. R., Giglou, A. N., & Gandomi, A. H. Microclimatic dynamics and hydrological patterns in urban heat islands—a comprehensive perspective. *Sustainable Cities Soc.* 10.1016/j.scs.2026.107184107184 (2026).

[43] Zanlorenzi, H. C. P., & da Silva Filho, D. F. O uso de barreiras vegetais para controle dos ventos em espaços abertos. *Revista IPT: Tecnologia e Inovação*, **3**(10) (2019). https://revista.ipt.br/revistaIPT/pt_BR/article/view/84

[44] Minatti, E., Ribeiro, A. A., Encina, C. C. C., Paranhos Filho, A. C. Análise multi-temporal de imagens de satélite e NDVI em unidade de conservação. *Res. Soc. Dev.* **12** (4), e1112440839. 10.33448/rsd-v12i4.40839 (2023).

[45] Barbosa, A. H. S., Carvalho, R. G., & Camacho, R. G. V. Aplicação do NDVI para a Análise da Distribuição Espacial da Cobertura Vegetal na Região Serrana de Martins e Portalegre—Estado do Rio Grande do Norte. *Revista do Departamento de Geografia*. **33**, 128 (2017).

[46] Brandão, D. O., Arieira, J. & Nobre, C. A. Impactos das mudanças climáticas na sociobioeconomia da Amazônia. *Estudos Avançados*, 38(112): pp. 249–270, (2024). 10.1590/s0103-4014.202438112.014

